# *Streptomyces marincola* sp. nov., a Novel Marine Actinomycete, and Its Biosynthetic Potential of Bioactive Natural Products

**DOI:** 10.3389/fmicb.2022.860308

**Published:** 2022-04-28

**Authors:** Songbiao Shi, Linqing Cui, Kun Zhang, Qi Zeng, Qinglian Li, Liang Ma, Lijuan Long, Xinpeng Tian

**Affiliations:** ^1^Key Laboratory of Tropical Marine Bio-Resources and Ecology, Chinese Academy of Sciences, Guangdong Key Laboratory of Marine Materia Medica, RNAM Center for Marine Microbiology, Sanya Institute of Oceanology, South China Sea Institute of Oceanology, Chinese Academy of Sciences, Guangzhou, China; ^2^Southern Marine Science and Engineering Guangdong Laboratory, Guangzhou, China; ^3^University of Chinese Academy of Sciences, Beijing, China

**Keywords:** *Strepomyces marincola* sp. nov., biosynthetic potential, polyphasic taxonomy, new bioactive secondary metabolites, marine actinobacteria

## Abstract

Marine actinomycetes are an important source of antibiotics, but many of them are yet to be explored in terms of taxonomy, ecology, and functional activity. In this study, two marine actinobacterial strains, designated SCSIO 64649^T^ and SCSIO 03032, were isolated, and the potential for bioactive natural product discovery was evaluated based on genome mining, compound detection, and antimicrobial activity. Phylogenetic analysis of the 16S rRNA gene sequences showed that strain SCSIO 64649^T^ formed a single clade with SCSIO 03032 (similarity 99.5%) and sister clades with the species *Streptomyces specialis* DSM 41924^T^ (97.1%) and *Streptomyces manganisoli* MK44^T^ (96.8%). The whole genome size of strain SCSIO 64649^T^ was 6.63 Mbp with a 73.6% G + C content. The average nucleotide identity and digital DNA–DNA hybridization between strain SCSIO 64649^T^ and its closest related species were well below the thresholds recommended for species delineation. Therefore, according to the results of polyphasic taxonomy analysis, the strains SCSIO 64649^T^ and SCSIO 03032 are proposed to represent a novel species named *Streptomyces marincola* sp. nov. Furthermore, strains SCSIO 64649^T^ and 03032 encode 37 putative biosynthetic gene clusters, and *in silico* analysis revealed that this new species has a high potential to produce unique natural products, such as a novel polyene polyketide compounds, two mayamycin analogs, and a series of post-translationally modified peptides. In addition, other important bioactive natural products, such as heronamide F, piericidin A1, and spiroindimicin A, were also detected in strain SCSIO 64649^T^. Finally, this new species’ metabolic crude extract showed a strong antimicrobial activity. Thanks to the integration of all these analyses, this study demonstrates that the novel species *Streptomyces marincola* has a unique and novel secondary metabolite biosynthetic potential that not only is beneficial to possible marine hosts but that could also be exploited for industrial applications.

## Introduction

In recent years, the search and discovery of novel microbes producing new active secondary metabolites have been urgently needed to counter and reverse the spread of new and emerging diseases and antibiotic-resistant pathogens in recent years (Payne et al., 2007). Actinobacteria are prolific producers of antibiotics and important suppliers to the pharmaceutical industry as they can produce a wide variety of secondary metabolites (van der Meij et al., 2017). Actinobacteria are also widely distributed throughout marine habitats, which, different from terrestrial habitats, are characterized by highly dynamic pressure, salinity, pH, dissolved oxygen, and light intensity. Marine actinobacteria have been attracting particular attention as new producers of novel antibiotics such as salinosporamide (Feling et al., 2003), ilamycin (Ma J. et al., 2017), and anticancer agents with unusual properties (Manivasagan et al., 2014a; Hassan and Shaikh, 2017; Davies-Bolorunduro et al., 2021). The genus *Streptomyces* alone accounts for a remarkable 80% of the actinobacterial natural products and, therefore, has unrivaled biosynthetic capacity in the microbial world (Manivasagan et al., 2014b; Hu et al., 2015). In particular, marine *Streptomyces* are known to produce broad-range-active natural products with immunosuppressant, antifungal, anticancer, antiparasitic, or antithrombotic activities (Ser et al., 2017; Wang et al., 2021), such as pactamides (Saha et al., 2017) and streptoseomycin (Zhang et al., 2019). Thus, the isolation and characterization of novel marine streptomycetes species are important for identifying new potential bioactive compounds.

The strain *Streptomyces* sp. SCSIO 03032 was isolated from a deep-sea sediment sample of the Indian Ocean in 2012. This strain was reported to produce α-pyridone antibiotics (piericidins A1/E1) (Chen et al., 2014), new polycyclic macrolactams (heronamides D–F) (Zhang et al., 2014a; Zhu et al., 2015), and unusual bisindole alkaloids (spiroindimicins A–D, G/H, indimicins A–G, and lynamicins A/D/F/G) (Zhang et al., 2012; Saurav et al., 2014; Zhang et al., 2014b; Ma L. et al., 2017; Liu et al., 2019) with excellent cytotoxic activity and antimicrobial activity. The intact biosynthetic gene clusters (BGCs) of piericidins (Chen et al., 2014) and heronamides (Zhu et al., 2015) and partial BGCs of spiroindimicins/indimicins/lynamicins (Ma L. et al., 2017; Liu et al., 2019) have also been reported from this strain. Interestingly, while investigating coral symbiotic microbial diversity, we isolated strain SCSIO 64649^T^, which was revealed to have 99.5% 16S rRNA gene sequence similarity with strain SCSIO 03032. This discovery was key as, while strain SCSIO 03032 is a producer of highly active compounds, its taxonomic status is undetermined. Therefore, this study was designed to establish the taxonomic status of strains SCSIO 64649^T^ and SCSIO 03032 using a polyphasic taxonomic approach. We identify the strains as one, same new species named *Streptomyces marincola* sp. nov. and evaluate its biosynthesis potential for novel natural product discovery through genome mining, compound detection, and antimicrobial activity evaluation.

## Materials and Methods

### Isolation and Maintenance

Strain SCSIO 64649^T^ was isolated from colonies of *Favites* sp. scleractinian corals collected at a depth of 2 m from the South China Sea off the Luhuitou Peninsula, Hainan Province, China (18.50°N, 109.46°E). The coral samples were washed with sterile natural seawater and then processed according to Zhou et al. (2020). The samples were diluted 100 times and plated onto 1/10 tryptic soy agar (TSA) prepared with natural seawater. After inverted culturing at 28°C for 15 days, strain SCSIO 64649^T^ was selected and purified by routine cultivation on 2216E medium at 28°C.

Strain SCSIO 03032 had been isolated using a modified ISP 2 medium from a deep-sea sediment sample collected at a depth of –3,412 m from the Bay of Bengal in the Indian Ocean (9.988°N, 87.995°E) (Zhang et al., 2012). Strains SCSIO 64649^T^ and SCSIO 03032 were preserved in glycerol suspensions (30%, v/v) at –80°C. The strain *S. specialis* DSM 41924^T^ was obtained from the Marine Culture Collection of China (MCCC) and cultured under the same conditions as the reference strain.

### Phylogenetic Analyses

Genomic DNA was extracted using a genomic DNA extraction kit (QIAGEN, Germany), and the amplification of the 16S rRNA sequence was carried out as previously described in Li et al. (2007). The identification and calculation of pairwise 16S rRNA gene sequence similarity were determined using EzBioCloud.^1^ Phylogenetic relationships were investigated using the neighbor-joining (Saitou and Nei, 1987), maximum-likelihood (Felsenstein, 1981), and maximum-parsimony methods (Fitch, 1971) on the MEGA 11 program with a bootstrap value of 1,000 resampling replicates (Felsenstein, 1985).

### Whole-Genome Sequencing, Analysis, and Biosynthetic Evaluation

The complete genome of strain SCSIO 64649^T^ was sequenced on a PacBio RS II platform by the Tianjin Biochip Corporation (Tianjin, China). *De novo* genome assembly was carried out following a hierarchical genome-assembly process (HGAP) (Chin et al., 2013), using HGAP4 (Pacific Biosciences, SMRT Link V6.0). The phylogenomic tree was reconstructed using 120 marker genes with the GTDB-Tk software toolkit (Chaumeil et al., 2019). The genomes were annotated by the Rapid Annotation using Subsystem Technology (RAST version 2.0) (Overbeek et al., 2014). Barrnap was used to predict rRNA information, and tRNAscan was used to predict the tRNAs (Chan and Lowe, 2019). CRISPR arrays and their associated proteins were searched by CRISPRCasFinder (Couvin et al., 2018). Prophage Hunter was used for finding active prophages from the whole genome (Song et al., 2019). Metabolic pathways in a single bacterium were reconstructed using the online tool KEGG Mapper (Kanehisa and Sato, 2020). The whole genome and orthologous genes among *Streptomyces* species were compared using OrthoVenn2 (Xu et al., 2019). Genomic island prediction was performed by using IslandViewer 4 (Bertelli et al., 2017). BGCs of secondary metabolisms were predicted in antiSMASH web service (version 6.0) (Blin et al., 2021). The compound structures were predicted based on genome sequence using PRISM4 (Skinnider et al., 2020). The average nucleotide identity (ANI) values were calculated using ChunLab’s online ANI calculator (Yoon et al., 2017). The estimated digital DNA–DNA hybridization (dDDH) values were calculated using the Genome-to-Genome Distance Calculator (GGDC 2.1), and Formula 2 was used as recommended for the calculation of dDDH (Meier-Kolthoff et al., 2013). The estimation of average amino acid identity (AAI) was determined using the tool AAI calculator.^2^

### Cultural and Phenotypic Properties

After incubation on ISP 2 at 28°C for 14 days, cell morphology was observed using a scanning electron microscope (Hitachi s-3400N). Cultural characteristics were tested on ISP 1, ISP 2, ISP 3, ISP 4, ISP 5, ISP 6, ISP 7 agar, R2A agar, Czapek’s agar, TSA, 2216E, and nutrient agar (NA) for 2 weeks at 28°C. The color of aerial and substrate mycelium and soluble pigments was determined using the ISCC-NBS color charts. Anaerobic growth was determined after 4 weeks of incubation at 28°C using the GasPak EZ Anaerobe Pouch Systems (BD). Growth at different temperatures, salinities, and pH was tested in ISP 2 broth as in Wang et al. (2018). Catalase activity was determined as the production of bubbles after the addition of 3% (v/v) hydrogen peroxide (H_2_O_2_). Tests for hydrolysis of starch, cellulose, gelatin, and Tweens (20, 40, 60, and 80) and H_2_S production, coagulation, and peptonization of milk were performed using the methods previously described (Gonzalez et al., 1978). Biochemical properties and enzyme activities were tested using API 20NE and API ZYM kits (bioMérieux, France) according to the manufacturer’s instructions. The ability to metabolize sole sources of carbon and nitrogen was tested with Biolog GEN III microplates. The susceptibility to antimicrobial agent was determined by the disk diffusion method (Bauer et al., 1966) with the following antibiotics (microgram per disk, Oxoid, United Kingdom): amikacin (30), amoxicillin (10), ampicillin (10), chloramphenicol (30), ciprofloxacin (5), erythromycin (15), gentamicin (10), lincomycin (2), neomycin (30), norfloxacin (10), novobiocin (5), penicillin G (10), rifampicin (5), streptomycin (10), kanamycin (30), tetracycline (30), tobramycin (10), and vancomycin (30).

### Chemotaxonomy

The cell biomass was collected for chemotaxonomic analyses after growing the strains on ISP 2 at 28°C for 1 week. Fatty acids from strains SCSIO 64649^T^ and SCSIO 03032 and the reference strain were extracted and analyzed using the standard protocol of the MIDI system (Sherlock version 6.1; database TSBA6). Polar lipids were examined and identified by two-dimensional TLC using silica gel 60 plates (Merck) with four dye agents (Minnikin et al., 1984). Menaquinones were extracted from freeze-dried biomass, purified, and analyzed by high-performance liquid chromatography (HPLC) (Collins et al., 1977) using an Agilent TC-C18 column (250 × 4.6 mm, 5 μm). The cell-wall diamino acid was analyzed from whole-cell hydrolyzates as previously described (Tang et al., 2009). For sugar analysis, cell walls were hydrolyzed in 0.5 M H_2_SO_4_ at 100°C for 2 h and analyzed by TLC on cellulose plates (Whiton et al., 1985).

### Identification of Bioactive Compounds

To identify the compounds produced by strains SCSIO 64649^T^ and SCSIO 03032, both strains were fermented in ISP 3 and ISP 4 media with 3% sea salt, in a 250 ml Erlenmeyer flask, and cultivated on a rotary shaker (200 rpm/min) at 28°C for 7, 9, and 11 days. For each fermentation sample, 5 ml was extracted with 10 ml butanone, and the crude extracts were dissolved with DMSO after evaporation. The extracts were analyzed by HPLC and LC-HR-MS after filtration through 0.2 μm syringe filters. Ten microliter per samples was injected on an Agilent 1260 HPLC equipped with a diode array detector (DAD) and Agilent TC-C18 Column (250 × 4.6 mm, 5 μm). The HPLC gradient was as follows: UV detection at 254 nm; solvent A, acetonitrile (10%) in water with formic acid (0.1%); solvent B, acetonitrile (90%) in water; 5–100% B (0–20 min); 100% B (20–21 min); 100%–5% B (21–22 min); and 5% B (22–30 min) with a flow rate of 1.0 ml/min. ESI-MS data were measured with an LCQ Deca XP HPLC/MS spectrometer (Bruker).

### *In vitro* Antimicrobial Activity Assay

The antimicrobial activity of crude extracts was evaluated by the agar well diffusion method against seven indicator microorganisms: *Staphylococcus aureus* ssp. *aureus* CGMCC 1.2386, *Bacillus subtilis* ssp. *spizizenii* CGMCC 1.1849, *Escherichia coli* CGMCC 1.2385, *Pseudomonas aeruginosa* DSM 50071, *Candida* albicans CGMCC 2.2086, *Aspergillus niger* CICC 2487, and *Micrococcus luteus* CGMCC 1.2299. The butanone extracts were dissolved in DMSO for antimicrobial activity detection. After incubating the plate at 28°C for 48 h, the antimicrobial activity was determined by the inhibition zone around the samples.

## Results and Discussion

### Phylogenetic Analyses

**FIGURE 1:**
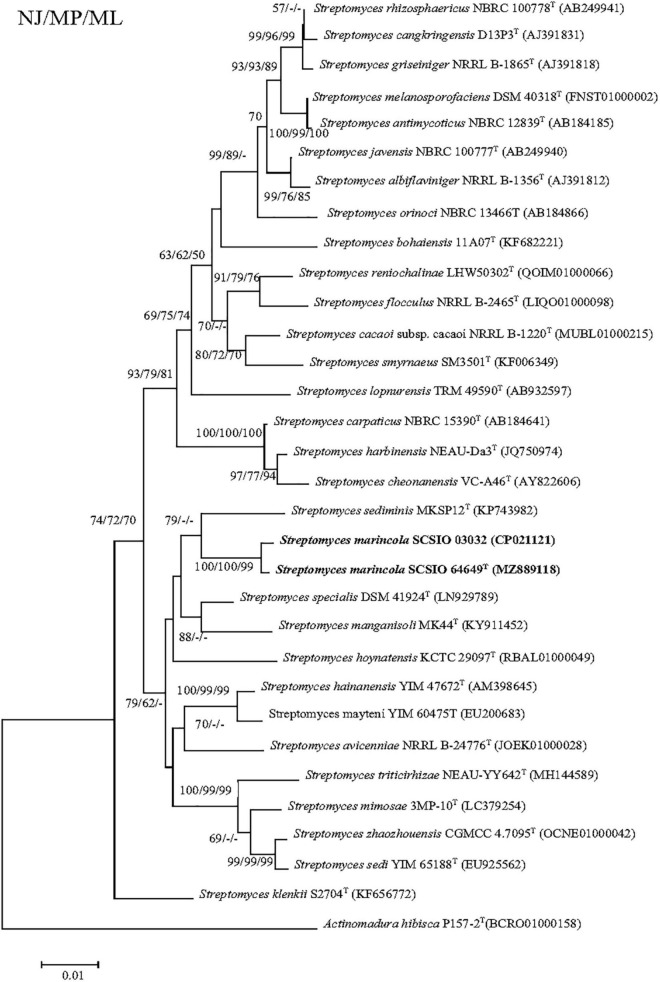
Neighbor-joining tree showing phylogenetic relationships between strains SCSIO 64649^T^, SCSIO 03032, and related *Streptomyces* species, based on 16S rRNA gene sequences. *Allostreptomyces psammosilenae* YIM DR4008^T^ (KX689228) was added as an outgroup. Bootstrap values are shown from left to right for neighbor-joining, maximum-likelihood, and maximum-parsimony trees based on 1,000 replications. Bar, 0.01 sequence divergence.

**FIGURE 2:**
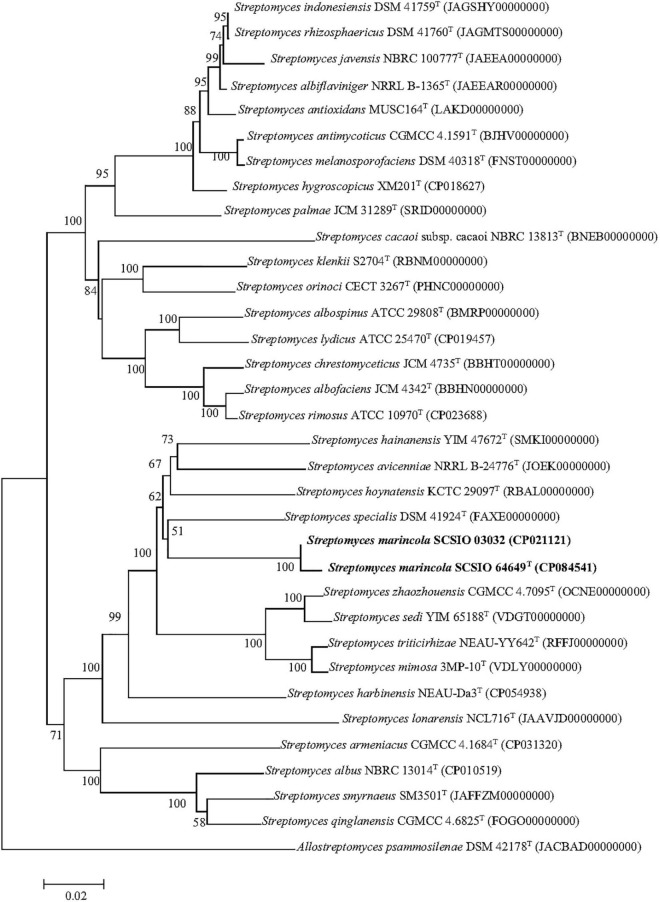
Phylogenetic analysis based on genome sequences of strains SCSIO 64649^T^, SCSIO 03032, and related *Streptomyces* species. The RAxML tree was calculated with the PhyloPhlAn software. *Allostreptomyces psammosilenae* YIM DR4008^T^ was added as an outgroup. Bar, 0.02 sequence divergence.

The nearly complete 16S rRNA gene sequence of strain SCSIO 64649^T^ was obtained (1,432 bp; GenBank accession number MZ889118). Based on 16S rRNA gene sequence comparison, strain SCSIO 64649^T^ showed a high sequence similarity with SCSIO 03032 (99.5%), the published species *S. specialis* DSM 41924^T^ (97.1%), *S. manganisoli* MK44^T^ (96.8%), and *S. sediminis* MKSP12^T^ (96.6%) and less than 96.5% similarity with other species. Likewise, strain SCSIO 03032 was most similar to the same type species with minor differences in the percentage similarity: *S. specialis* DSM 41924^T^ (97.7%), *S. sediminis* MKSP12^T^ (97.1%), and *S. manganisoli* MK44^T^ (97.0%). The 16S rRNA gene similarity between SCSIO 64649^T^ and SCSIO 03032 is higher than the threshold of 98.65% for differentiating two species (Kim et al., 2014), while the similarities between these two strains with other known species are below this threshold, therefore supporting the idea that the strains represent the same species. Phylogenetic analysis showed that the strains SCSIO 64649^T^ and SCSIO 03032 clustered together with *S. sediminis* MKSP12^T^ and formed a separate phylogenetic branch parallel with *S. specialis* DSM 41924^T^ and *S. manganisoli* MK44^T^. This topology was supported by all three algorithms employed (Figure 1). The phylogenomic tree showed that strain SCSIO 64649^T^ stably clustered with SCSIO 03032 and their nearest-neighbor, *S. specialis* DSM 41924^T^ (Figure 2). Strain SCSIO 64649^T^ showed an ANI value of 80.2%, a dDDH value of 23.8%, and an AAI value of 74.5% with *S. specialis* DSM 41924^T^ and was followed by *S. hoynatensis* KCTC 29097^T^ (79.0, 22.4, and 73.3%, respectively) (Table 1). Nearly identical values were observed between strain SCSIO 03032 and *S. specialis* DSM 41924^T^. The lower values of ANI, dDDH, and AAI between these two strains and other closely related *Streptomyces* species are also shown in Table 1. These values are all far from the recommended similarity thresholds (ANI < 95–96%, dDDH < 70%, and AAI < 95%) (Richter and Rossello-Mora, 2009; Meier-Kolthoff et al., 2013; Konstantinidis et al., 2017). However, strains SCSIO 64649^T^ and SCSIO 03032 revealed high ANI, dDDH, and AAI similarity (96.6, 84.9, and 96.4%, respectively) and therefore fall within the recommended thresholds. Given all this evidence, we suggest that strains SCSIO 64649^T^ and SCSIO 03032 represent the same novel species in the genus *Streptomyces*.

**TABLE 1:** ANI, dDDH values, and AAI found between isolates and their closest species.

Strains	ANI (%)		dDDH (%)		AAI (%)	
	SCSIO 64649^T^	SCSIO 03032	SCSIO 64649^T^	SCSIO 03032	SCSIO 64649^T^	SCSIO 03032
SCSIO 64649^T^	100	96.6	100	84.9	100	96.4
SCSIO 03032	96.6	100	84.9	100	96.4	100
*S. specialis* DSM 41924^T^	80.2	80.3	23.8	23.7	74.5	74.7
*S. hoynatensis* KCTC 29097^T^	79.0	79.0	22.4	22.5	73.3	73.3
*S. hainanensis* YIM 47672^T^	79.2	79.2	22.8	22.9	72.8	73.1
*S. klenkii* KCTC 29202^T^	76.5	79.0	21.2	21.2	65.2	65.2

### Genome Characteristics

The complete genome of strain SCSIO 64649^T^ was composed of one linear chromosome (6,629,020 bp, GenBank accession number CP084541) with G + C content of 73.6%, 5,774 genes, 5,567 protein-coding genes, 133 pseudo genes, 60 tRNA genes, and 15 rRNA genes (5 23S, 5 5S, and 5 16S) (Supplementary Table 1). The genome of strain SCSIO 03032 has been previously described (Ma et al., 2021). The 16S rRNA gene sequences of SCSIO 64649^T^ obtained from its genome and Sanger sequencing method showed 99.3% sequence similarity. Eleven and seven genomic islands were identified in the genome of strains SCSIO 64649^T^ and SCSIO 03032, respectively (Supplementary Table 2). Evidence of genomic islands linking secondary metabolism to functional adaptation has been provided in marine actinobacteria *Salinispora* (Penn et al., 2009), which may explain the cosmopolitan distribution of SCSIO 64649^T^ and SCSIO 03032. Three prophage-like sequences were identified in genomes of strains SCSIO 64649^T^ and SCSIO 03032, and one out of three was active. The closest elements related to *Mycobacterium* phage Hammy were found in SCSIO 64649^T^, while the *Xanthomonas* phage Xoo-sp2 was found in SCSIO 03032 (Supplementary Table 3). A total of 37 secondary metabolite BGCs with eight new clusters were discovered in strains SCSIO 64649^T^ and SCSIO 03032 genome sequences. These secondary metabolite BGCs mainly covered polyketide synthase (PKS), terpene, siderophore, non-ribosomal peptide synthase (NRPS), thiopeptide, lanthipeptide, lasso peptide, indole, and 15 unknown gene clusters. The BGC maps of strains SCSIO 64649^T^ and SCSIO 03032 were drawn (Supplementary Figure 1). Strain SCSIO 64649^T^ encoded for 10 CRISPR arrays and 15 Cas-proteins, including Cas1-6, Csh2, and the Cse3-5 family. A novel knock-in CRISPR-based approach introducing the kasO*p promoter cassette to drive expression of putative BGCs was successfully used for *S*. *viridochromogenes* (Zhang M. M. et al., 2017), leading to expression and production of novel secondary metabolites. Since the presence of putative CRISPR arrays and all known cascade proteins in two genomes implicates the activity of the CRISPR/Cas immune system in the two strains, in the future, the activation of this species’ silent and unusual BGCs could be possible using the above-mentioned CRISPR-based tools.

### Phenotypic Characteristics

**FIGURE 3:**
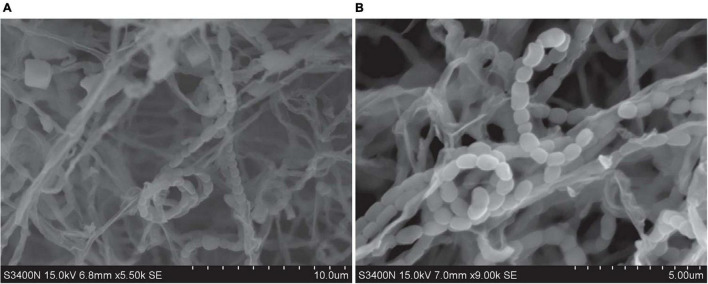
Scanning transmission electron microscope images show the cell morphology of strains SCSIO 64649^T^ **(A)** and SCSIO 03032 **(B)** after incubation on ISP 2 medium for 14 days at 28°C.

Strains SCSIO 64649^T^ and SCSIO 03032 are Gram-stain-positive and aerobic actinomycetes with extensively branched substrate mycelia and aerial hyphae, which differentiate into spiral spore chains consisting of elliptical or short-rod spores (∼1.0–1.3 × 0.7–0.9 μm) with smooth surfaces (Figure 3). The strains grow well on ISP 2, ISP 4, ISP 7, NA, and 2216E and moderately well on ISP 1, ISP 3, ISP 5, ISP 6, TSA, and CA media (Supplementary Table 4 and Supplementary Figure 2). The colors of the aerial and substrate mycelia are media dependent. The diffusible melanin is observed only on the ISP 2 medium. Growth of strain SCSIO 64649^T^ occurs at 15–40°C (optimal 28°C), pH 6–9 (optimal 7–8), and up to 9% NaCl (optimal 4%), different from *S. specialis* DSM 41924^T^ in growth conditions (Table 2). Strain SCSIO 64649^T^ can be easily distinguished from the type strains of its closest neighbors through its phenotypic properties (Table 2 and Supplementary Table 5).

**TABLE 2:** Phenotypic properties that distinguish strains SCSIO 64649^T^ and SCSIO 03032 from their closest phylogenomic relatives.

Characteristics	SCSIO 64649^T^	SCSIO 03032	*S. specialis* DSM 41924^T^	*S. manganisoli* MK44^T^*
**Sources**	**Stony coral**	**Sediment**	**Soil**	**Soil**
pH range	6.0–9.0	6.0–9.0	6.0–10.0	5.0–9.0
Temperature range (°C)	15–40	15–40	25–40	10–40
NaCl range (%)	0–9	0–5	0–7	0–5
Gelatin liquefaction	+	+	+	–
Hydrolysis of aesculin	+	+	–	–
Carbon source utilization				
Myo-Inositol	–	+	+	–
D-Arabinose	–	+	+	+
Cellobiose	–	+	–	+
D-Fructose	–	+	–	–
D-Galactose	+	–	–	–
D-Mannose	–	–	–	+
D-Ribose	–	–	+	–
D-Rhamnose	–	+	–	+
Lactose	–	–	+	+
Maltose	+	+	–	+
Xylitol	–	–	+	–
G+C content (%)	73.6	73.5	72.8	75.7

All strains are positive for hydrolysis of starch and Tweens 40 and 80 but negative for nitrate reduction, H_2_S production, milk coagulation, and peptonization. All data come from this study (except data marked with *). +, positive; –, negative.

*Data from Mo et al. (2018).

### Chemotaxonomic Analyses

The cellular fatty acids of strain SCSIO 64649^T^ detected (>10%) were iso-C_16:0_ (37.7%) and C_12:0_ (13.1%). While the major fatty acids of *S. specialis* DSM 41924^T^ are iso-C_16:0_ (33.2%) and anteiso-C_17:0_ (19.3%). And also the difference between them is in the amounts of C_18:3_ ω6*c*, iso-C_16:1_ H, etc. (Table 3). The polar lipid profiles of strain SCSIO 64649^T^ comprised diphosphatidylglycerol, phosphatidylglycerol, phosphatidylethanolamine, phosphatidylinositol mannoside, phosphatidylinositol, glycerol lipid, and six unidentified phospholipids. Strain SCSIO 64649^T^ differed from *S. specialis* DSM 41924^T^ in its polar lipid composition by having four unidentified phospholipids (PL3-6) and missing unidentified lipids (L1-2) (Supplementary Figure 3). *LL*-2,6-Diaminopimelic acid was identified as the cell-wall diamino acid of strains SCSIO 64649^T^ and DSM 41924^T^. The cell sugars identified in both strains were galactose, glucose, xylose, and ribose. The predominant menaquinone of strain SCSIO 64649^T^ was MK-10(H_4_) (75.5%) and MK-10(H_6_) (16.1%), and minor amounts (<3%) of MK-9(H_4_), MK-9(H_6_), and MK-10 (H_8_) were also detected. All the above menaquinones were also detected in *S*. *specialis* DSM 41924^T^; however, the proportions of MK-10(H_4_) and MK-10(H_6_) were different (79.6 and 16.0%, respectively).

**TABLE 3:** Cellular fatty acid profiles of strain SCSIO 64649^T^ and its neighbors in genus *Streptomyces*.

Fatty acid	SCSIO 64649^T^	SCSIO 03032	*S. specialis* DSM 41924^T^	*S. manganisoli* MK44*^T&^*
C_9:0_	2.0	ND	TR	ND
C_12:0_	**13.1**	ND	2.8	1.0
Iso-C_15:0_	2.3	1.0	2.8	2.0
Anteiso-C_15:0_	2.2	TR	1.9	1.0
C_15:1_ ω6*c*	1.1	TR	TR	0.5
Iso-C_16:1_ G	ND	**25.2**	ND	**14.4**
Iso-C_16:1_ H	TR	ND	6.0	ND
Iso-C_16:0_	**37.7**	**43.2**	**33.2**	**51.3**
C_16:0_	6.2	1.5	2.6	ND
Anteiso-C_17:1_ ω9*c*	5.2	5.5	9.2	4.4
Iso-C_17:0_	2.0	1.9	3.6	1.6
Anteiso-C_17:0_	7.3	6.2	**19.3**	**7.0**
C_17:0_ cyclo	5.1	1.0	1.7	0.4
Iso-C_18:0_	TR	2.0	3.6	0.2
Iso-C_18:1_ H	1.2	1.9	TR	1.2
C_18:3_ ω6*c*	5.6	ND	TR	ND
Sum in feature 3	2.1	1.9	4.3	3.4
Sum in feature 9	2.7	2.5	5.4	8.6

*Strains: 1, SCSIO 64649^ T^; 2, SCSIO 03032; 3, S. specialis DSM 41924^ T^; 4, S. manganisoli MK44^ T^. All data from this study except for S. manganisoli MK44^ T^; cells were collected after incubation on 2216E at 28°C for 7 days. The major fatty acids (greater than 10%) are shown in bold. TR, less than 1%; ND, not detected. Summed feature 3 comprises C_16:1_ ω6c and/or C_16:1_ ω7c. Summed feature 9 comprises iso-C_17:1_ ω9c and 10-methyl C_16:0_.*

*^&^Data from Mo et al. (2018).*

### Comparative Genome Analysis

**FIGURE 4:**
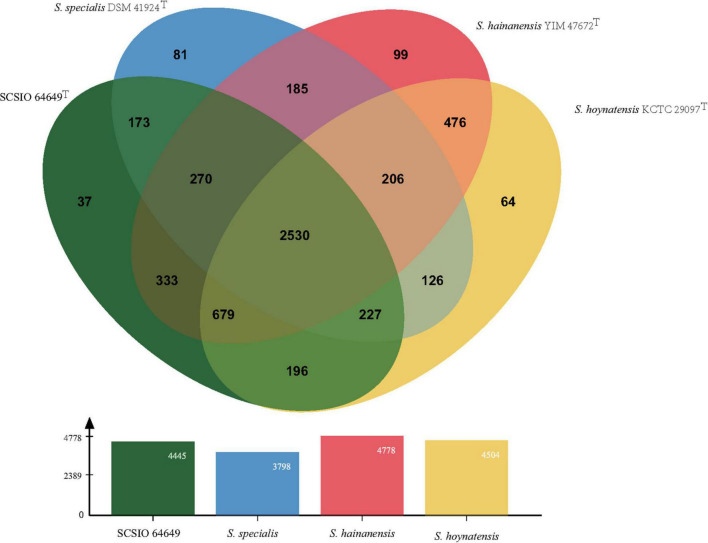
Venn diagram representing the core orthologs and unique genes for strain SCSIO 64649^T^ and closely related type strains.

**FIGURE 5:**
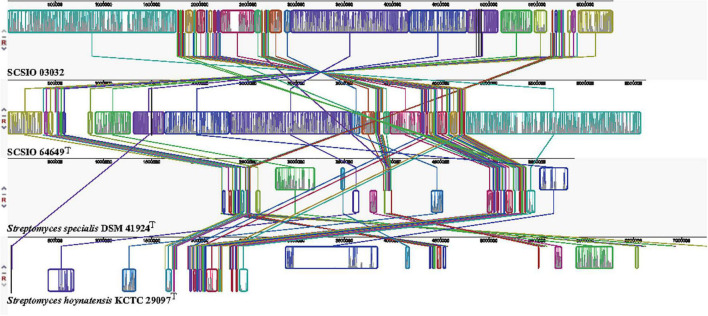
Genome alignment between strains SCSIO 64649^T^, SCSIO 03032, *S. specialis* DSM 41924^T^, and *S. hoynatensis* KCTC 29097^T^.

The core genes and specific genes of the two isolates and the species *S. specialis* DSM 41924^T^ and *S. hoynatensis* KCTC 29097^T^ were determined. With OrthoVenn2, a total of 2,676 core genes were found in the four strains (Figure 4), and 10 genes were unique to SCSIO 64649^T^. These genes encoded functional minor molecules, such as oxidoreductase, transferase, and hydrolase activity. A comparison of the orthologous gene numbers revealed that SCSIO 64649^T^ shares 90.5% (4,748/5,249) sequence similarity with SCSIO 03032 and a lower proportion with *S. specialis* DSM 41924^T^ (63.0%) and *S. hoynatensis* KCTC 29097^T^ (60.4%). These results help to separate strains SCSIO 64649^T^ and SCSIO 03032 from closely related type strains and identify them as the same, novel species. For a comprehensive genome comparison, a synteny block analysis was performed on highly conserved large segment sequences in strains SCSIO 64649^T^, SCSIO 03032, *S. specialis* DSM 41924^T^, and *S. hoynatensis* KCTC 29097^T^ by a progressive mauve tool. Although the four strains shared many locally collinear blocks with their reference genomes, all exhibited large-scale genome rearrangement (Figure 5). The positions of the locally collinear blocks highlight the complex evolutionary history of these strains. A total of 37 putative secondary metabolite secondary metabolite BGCs were detected in strains SCSIO 64649^T^ and SCSIO 03032 (Supplementary Table 6). The BGCs identified share homology to 22 known gene clusters with known metabolic products, such as the compatible solute ectoine, siderophore desferrioxamine B, the carotenoid light-harvesting pigment isorenieratene, and terpene hopene. In addition, SCSIO 64649^T^ contained some unique gene clusters, including one encoding for ketomemicin B3/B4 (Kawata et al., 2017). The BGCs of DSM 41924^T^ and KCTC 29097^T^ were also analyzed, and the type and number of coding gene clusters were markedly different from strains SCSIO 64649^T^ and SCSIO 03032 (Supplementary Figure 4). These results further illustrate the diverging metabolic potential of this new *Streptomyces* species.

### Biosynthetic Potential of the New *Streptomyces* Species

To evaluate the secondary metabolite biosynthetic potential of the new species, the genomes of strains SCSIO 64649^T^ and SCSIO 03032 were analyzed with antiSMASH. A total of 32 and 29 putative secondary metabolite BGCs were detected and accounted for 19.7 and 14.4% of their genomes, respectively. This analysis revealed that there is a higher percentage of the genome dedicated to secondary metabolites in this species compared with the representative actinomycetes strain *S. coelicolor* (4.5%) (Cimermancic et al., 2014). Strain SCSIO 64649^T^ comprises 10 different types of BGCs, including those coding for ribosomally synthesized and post-translationally modified peptides (RiPPs) (thiopeptide, lanthipeptide, lasso peptide, RRE-containing, and RiPP-like), PKS, NRPS, indole, terpene, siderophore, guanidinotides, melanin, ectoine, and phenazine (Supplementary Table 6). In addition to the BGCs encoding for common secondary metabolites produced by genus *Streptomyces*, such as desferrioxamine E, melanin, and hopene, 11 BGCs showed no similarity to any reference BGCs, and nine BGCs had 60% of genes with high similarity to homologs from known BGCs. This highlights the potential of SCSIO 64649^T^ to produce novel secondary metabolites.

A remarkable feature of the strain SCSIO 64649^T^ genome is the presence of nine PKS-coding BGCs, encoding three different kinds of PKS (six PKS I, two PKS II, and one PKS III). The six PKS I clusters (#3, #4, #9, #13, #15, and #30) showed variable similarity with reported BGCs (6–100%). Clusters #3 and #13 showed 100% similarity with BGCs encoding for piericidin A1 and heronamide F, which have been reported in SCSIO 03032 by Chen et al. (2014) and Zhu et al. (2015), respectively. SCSIO 64649^T^ presented intact BGCs of piericidin A1 and heronamide F. However, the predicted constructs of gene clusters 3# and 13# are quite different from piericidin A1 and heronamide F (Supplementary Figure 5) clarified in strain SCSIO 03032. *In silico* analysis of cluster #30 revealed low similarity with candicidin (23%, MIBiG accession number BGC0000034), which is a member of polyene polyketides possessing a series of conjugated unsaturated double bonds and exhibiting potent activities against fungal pathogens (Zhang L. et al., 2017; Sun et al., 2018). The polyketide backbone of candicidin is characterized by seven conjugated double bonds and is constructed by 21 PKS modules through condensing a *p*-aminobenzoic acid starter unit, 4 propionate molecules, and 14 acetate units (Chen et al., 2003). Detailed bioinformatics analysis revealed that the five core PKS genes (ctg1_5542-5546) in cluster 30# encode 18 modules, which were predicted to be responsible for condensation of seven propionates and 11 acetate units to form a polyketide backbone with three conjugated double bonds. In addition to the core PKSs, the post-PKS tailoring enzymes also showed much lower homologous similarity compared with candicidin coding sequences. Collectively, the difference in the core PKSs and the low sequence similarity of the post-PKS tailoring enzymes strongly indicated that cluster #30 encodes a new polyketide product. The predicted structure of the compound is shown in Supplementary Figure 5. Clusters #4 and #15 displayed low similarity with known gene clusters salinomycin (6%, MIBiG accession number BGC0000144) and stambomycins (16%, MIBiG accession number BGC0000151). This potential to produce novel metabolites, which cannot be predicted with bioinformatics analyses, needs to be confirmed by further secondary metabolite separation.

The type II PKS genes clusters #5 and #20 showed, respectively, 68 and 54% similarities with the mayamycin BGC (MIBiG accession number BGC0001661) from *Streptomyces* sp. 120454 (Bo et al., 2018). Mayamycin is a member of the angucycline-type polycyclic compounds that predominantly display anticancer and antibacterial activity and feature a tetracyclic benz[a]anthracene scaffold, which is derived via successive decarboxylative Claisen condensations of an acetyl-CoA starter unit and nine malonyl-CoA extender units (Kharel et al., 2012). Six PKS genes in BGC #5, encoding ketoacyl synthase, chain length factor, acyl carrier protein, two cyclases, and one ketoreductase, showed high similarity to the corresponding PKS enzymes (May 12–17) from the mayamycin gene cluster (Bo et al., 2018). This suggests the formation of angular tetracyclic rings in its biosynthetic pathway. Although the core PKS enzymes in BGC #5 showed high similarity to the homologs from angucycline-type BGC, the genes responsible for sugar biosynthesis are different from those involved in the amino sugar biosynthetic pathway of mayamycin. The mayamycin BGC contains six genes (*may5*, *6*, *7*, *9*, *10*, and *22*) encoding NDP-glucose phosphate nucleotidyltransferase, NDP-hexose 4,6-dehydratase, NDP-deoxyglucose-2,3-dehydratase, NDP-deoxyhexose 3-aminotransferase, NDP-4-keto-6-deoxyhexose reductase, and *N*-methyl transferase for the construction of amino sugar (Bo et al., 2018). The sequence analysis of BGC #5 revealed five open reading frames (ORFs) that could potentially be involved in the amino sugar biosynthetic pathway; these five ORFs are LC193_03460, 03465, 03470, 03480, and 03485, encoding for NDP-glucose phosphate nucleotidyltransferase, NDP-hexose 4,6-dehydratase, NDP-deoxyglucose-2,3-dehydratase, NDP-deoxyhexose aminotransferase, and dTDP-4-dehydrorhamnose-3,5-epimerase, respectively. This suggests the biosynthesis of novel mayamycin analogs with different amino sugars (Supplementary Table 7), and its predicted structure is shown in Supplementary Figure 5. Cluster #20 showed 54% similarity with the mayamycin BGC, indicating that this cluster also produces angucycline-type polycyclic compounds. However, the lower amino sequence similarity and different organization (Supplementary Table 8) indicate that cluster #20 may synthesize a novel mayamycin analog (Supplementary Figure 5), which is different from the product of cluster #5.

Nine BGCs are involved in the biosynthesis of RiPPs (thiopeptide, lanthipeptide, lasso peptide, RRE-containing, and RiPP-like). Only cluster #10 of nine RiPPs BGCs showed a high similarity (80%) to class III lanthipeptide of AmfS, which comprises biological surfactants that positively regulate the formation of aerial mycelia (Ueda et al., 2002). In the remaining eight RiPP BGCs, two BGCs (#8, #28) showed low similarities (<50%) to the known BGCs, and six BGCs (#10, #14, #17, #23, #24, and #29) did not match with known gene clusters. These findings revealed that strain SCSIO 64649^T^ has the potential to produce the novel RiPPs.

The remaining clusters in SCSIO 64649^T^, #16, #19, #21, and #26, are terpene BGCs, assumed to be similar to the BGCs of isorenieratene, geosmin, carotenoid, and hopene, respectively. Except for cluster #19, which shows 100% BGC similarity with geosmin, the other three clusters, #16 (37%), #21 (27%), and #26 (30%), showed low similarities with known BGCs, indicating that the strain also has the potential to produce novel terpene compounds.

Phage-encoded serine integrases are powerful tools for molecular genetics, because they can catalyze site-specific integration of DNA into bacterial host chromosomes in a highly controllable and predictable way (Gao et al., 2020). Twenty-one serine and subtilisin-like serine integrase genes were also annotated in the SCSIO 64649^T^ genome (Supplementary Table 9), and they belong to a family of proteins known to play several different biological roles (Karlsson et al., 2007). Further research on these specific proteases may also be relevant from an industrial perspective. Through the analysis of synthetic pathways by KEGG, we also found that, in addition to natural products, this new species has the potential to produce a variety of cofactors and vitamins, such as riboflavin, biotin, and VB12 (cobalamin). These results indicate that this new species offers the potential to discover novel natural products.

### Identification of Bioactive Compounds and Antimicrobial Activity Assay

**FIGURE 6:**
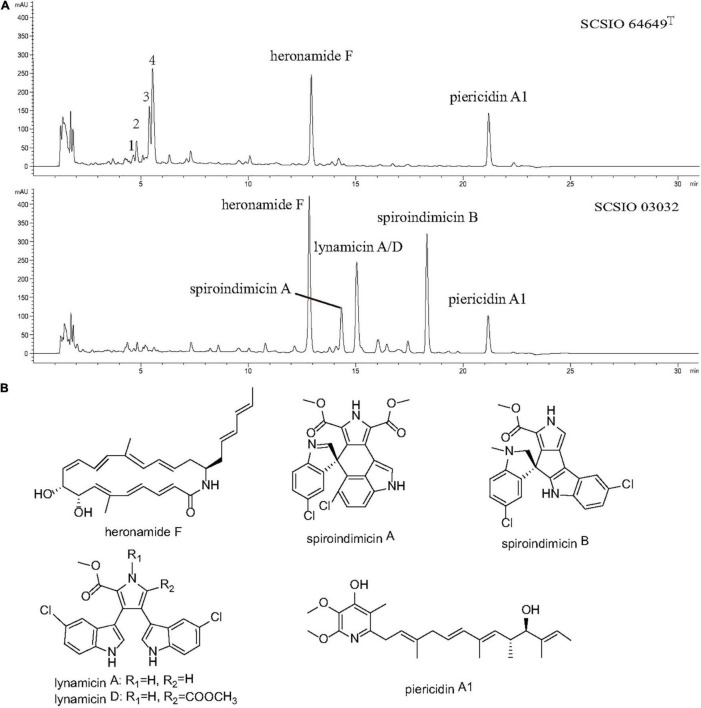
**(A)** LC-DAD isoplot of the analyzed extract showing compounds produced. **(B)** Structural formula of compounds isolated from strains SCSIO 64649^T^ and SCSIO 03032.

Strain SCSIO 03032 has been demonstrated to be able to produce five categories of bioactive compounds (piericidins, heronamides, spiroindimicins, indimicins, and lynamicins) under laboratory culture conditions (Zhang et al., 2012; Chen et al., 2014; Zhang et al., 2014a,b; Zhu et al., 2015; Ma L. et al., 2017; Liu et al., 2019). To identify the bioactive compounds produced by strain SCSIO 64649^T^, both strains were fermented using ISP 3 and ISP 4 media with the same conditions. The fermentation extracts were analyzed using LC-HR-MS and then compared. When fermented with ISP 4 medium, strain SCSIO 03032 produced four types of compounds (heronamide F, piericidin A1, spiroindimicins A and B, and lynamicins A/D); however, strain SCSIO 64649^T^ was found to only produce heronamide F and piericidin A1 as evidenced by ESI-MS data (422.2706 [M + H]^+^ and 416.2722 [M + H]^+^, respectively) (Figure 6A and Supplementary Figure 6). A yield of spiroindimicin B was detected in SCSIO 03032 but not detected in strain SCSIO 64649^T^ under the same fermentation conditions. The gene cluster coding for spiroindimicins in SCSIO 64649^T^ showed similar genetic organization, and the amino acid sequence of eight genes had a high degree of homology with a similarity greater than 90% with SCSIO 03032 (Supplementary Table 10). The inactivation of the spiroindimicin BGC in SCSIO 64649^T^ may be attributed to the unsuitability of the ISP 4 medium to produce spiroindimicin B. Indeed, when the strain SCSIO 64649^T^ was fermented in ISP 3 medium, spiroindimicin A was also readily detected (Supplementary Figure 7). Although the two strains belong to the same species, their secondary metabolites could be different because of their adaptations to different natural environments.

Piericidin A1 is a member of the piericidin family and features a 4-pyridinol core linked to a methylated polyketide side chain (Liu et al., 2012; Zhou and Fenical, 2016). A recent study revealed that piericidin A1 shows anti-renal cell carcinoma activity, which has laid the foundation for the development of new anti-kidney cancer drugs (Zhou et al., 2019). Heronamides D–F were isolated from strain SCSIO 03032 (Zhang et al., 2014a). Heronamides are a rare class of polyketide 20-membered macrolactams featuring unprecedented carbon/heteroatom skeletons that show potent antifungal metabolites and are produced by marine-derived actinomycetes (Raju et al., 2010). The spiroindimicins are a unique class of chlorinated indole alkaloids characterized by featuring unique [5,5] or [5,6] spiro-rings, which displayed moderate cytotoxicity against several cancer cell lines. The discovery of these bioactive compounds in strains SCSIO 64649^T^ and SCSIO 03032 suggests that this new species is an important source of piericidins, heronamides, and spiroindimicins.

Interestingly, four new peaks were found in extracts of strain SCSIO 64649^T^ fermented with ISP 4 medium. These appeared within 4–6 min with a molecular weight of 190.1226, 179.1171, 196.1691, and 208.1335 (Figure 6), but they were not detected in the fermentation extracts of strain 03032. Future research should focus on large-scale fermentation and separation to obtain more metabolites and extend the detection range of biological activity.

The fermentation extract of strain SCSIO 64649^T^ was also subjected to an antimicrobial activity assay to assess its potential to produce antimicrobial compounds. The analysis revealed that the fermentation extracts showed strong antimicrobial activity against *Aspergillus niger* and weak activity against *Micrococcus luteus* from two different fermentation media, but no antimicrobial activities to the other five indicative microorganisms (Supplementary Figure 8). It was also revealed that the fermentation extract on the ninth day showed better antimicrobial activity than that on the 7th and 11th days, which indicated that the production of active natural products is related to fermentation time. Although heronamide F has been reported to possess antifungal activity against *Candida albicans*, we detected only antifungal activity against *Aspergillus niger*. Therefore, it was deduced that strains SCSIO 64649^T^ and SCSIO 03032 produced different antifungal compounds. Results from genome mining and antimicrobial activity detection indicate that this new species has the great potential to produce novel natural products with potent antimicrobial activities.

### Ecological Role

Genome annotation and analysis revealed that this new species encodes many Eukaryotic-like proteins (ELPs), such as tetratricopeptide repeats, ankyrin repeats, and WD-40 proteins. These molecules may mediate bacterial–host interactions and modulate the host’s behavior (Reynolds and Thomas, 2016; Robbins et al., 2019). Four WD-40 repeats and one tetratricopeptide repeat were discovered in SCSIO 64649^T^, which indicates that it may have the potential to form symbiotic relationships with coral hosts. In fact, bacteria in sponges express ankyrin genes to avoid phagocytosis becoming residents (Jahn et al., 2019). Although strain SCSIO 03032 was isolated from deep-sea sediment, 18 tetratricopeptide repeats, 2 ankyrin repeats, and 1 WD-40 repeat were also discovered in its genome, suggesting a possible role for these motifs in maintaining symbiotic associations. ATP-binding cassette (ABC) transporters are ATP-dependent protein complexes, which are vital in mediating the transport of both organic and inorganic molecules across cell membranes (Denecke et al., 2021). A total of 240 ABC transporters and 144 ABC transporters were annotated in the genomes of SCSIO 64649^T^ and SCSIO 03032, respectively. These are thought to be involved in nutrient acquisition and to help maintain osmotic balance in the cell. In addition, strain SCSIO 64649^T^ has genes encoding for cobalt and zinc resistance as well as genes for copper oxidase, which are stress genes associated with osmotic and oxidative processes. VB12 plays an important role as an essential co-factor in various biochemical processes, is only produced by some bacteria and archaea, and requires more than 30 enzymes for the *de novo* synthesis (Banerjee and Ragsdale, 2003). Strains SCSIO 64649^T^ and SCSIO 03032 possess complete synthetic pathway genes and synthesis ability of VB12 by aerobic routes; therefore, they have the potential to supply VB12 for their hosts, which cannot produce it themselves. The antimicrobial activity of this new species may also protect the host against pathogens and predators. In return, the host may provide shelter from predators and adverse conditions to these microorganisms residing in its tissues and supply the essential nutrients for their growth and metabolism. All these features indicate that strain SCSIO 64649^T^ may be a beneficial microorganism for the coral holobiont thanks to its versatility and high adaptability to harsh environmental conditions.

## Discussion

There is a recognized positive correlation between the isolation and discovery of new actinomycetes and novel bioactive compound discovery. The present study was designed to establish the taxonomic status of this novel species and, further, to describe its biosynthesis potential to produce novel natural products through genome mining, compound detection, and antimicrobial activity analysis.

Morphological, phylogenetic, chemotaxonomic, and genomic analyses indicated that strains SCSIO 64649^T^ and SCSIO 03032 belong to the genus *Streptomyces*. Extensive analyses revealed that they are very different from their closest relatives *S. specialis* DSM 41924^T^ and *S. manganisoli* MK44^T^ in physiological, biochemical, and chemotaxonomic properties (Tables 2, 3 and Supplementary Table 5). Based upon these results and the ANI, dDDH, and AAI values, strains SCSIO 64649^T^ and SCSIO 03032 were found to represent the same novel species in genus *Streptomyces*, for which the name *Streptomyces marincola* sp. nov. is here proposed, with the type strain SCSIO 64649^T^.

A high percentage of this new species genome is dedicated to secondary metabolite production, as indicated by the length of the BGC-related sequences. Thirty-two secondary metabolite BGCs in strain SCSIO 64649^T^ were distributed across 10 different types. Eleven of them show no similarity to any reference BGCs, while nine BGCs representing 60% of related genes showed high similarity to homologs from known BGCs. This indicates that this novel species has the potential to produce new secondary metabolites. Detailed analysis of the PKS-coding BGCs and RiPPs revealed that the new species has potential for the biosynthesis of a novel polyene polyketide compound, two mayamycin analogs, and a series of RiPPs.

Three remarkable bioactive secondary metabolites were detected from SCSIO 64649^T^ as compared with 22 compounds from strain SCSIO 03032 found in a previous study. Most of these compounds showed multiple biological activities, which suggests that this new species could be an important source of useful compounds for the medical and agricultural industries. Extending the detection range of biological activity can improve the application of these compounds, such as piericidin A1. However, in this study, the putatively novel compounds were not further characterized, either because of unsuitable fermentation conditions or lack of expression. In addition, this new species showed strong antimicrobial activity, which was inconsistent with the activity of known compounds, indicating that different, unknown antifungal compounds were produced.

In conclusion, a “new species owns the novel genes, which relate the novel natural products”; this new *Streptomyces* species shows great potential to produce novel natural products that could be used by the medical and agricultural industries. Since, in recent years, several approaches for activating the BGCs have been developed (Liu et al., 2020, 2021; Nguyen et al., 2020), future studies should focus on not only the discovery of the uncultured or new microorganisms but also how to isolate more bioactive products with new methods and also to use the metabolic profiling in species-level systematics research.

### Descriptions of *Streptomyces marincola* sp. nov.

*Streptomyces marincola* (ma.rin’co.la. L. n. *mare* the sea; L. n. *incola* inhabitant; N.L. n. *marincola* inhabitant of the sea).

Gram-stain-positive and aerobic actinomycete that forms an extensively branched substrate mycelium and aerial hyphae that differentiate into spiral spore chains consisting of elliptical or short rod spores with smooth surfaces and grows well on ISP 2, ISP 4, ISP 7, NA, and 2216E media. The colors of the aerial and substrate mycelium are media dependent. The diffusible melanin is only observed on ISP 2 medium. Growth occurs at 15–40°C (optimal 28°C), at pH 6–9 (optimal pH 7–8), and up to 9% NaCl (optimal 4%). It is catalase and oxidase negative and positive for hydrolysis of gelatin; aesculin, Tweens 20, 40, 60, and 80; and starch, but negative for milk coagulation and peptonization, nitrate reduction, hydrolysis of cellulose, and H_2_S production. It is positive for lipase (C14), leucine arylamidase, naphthol-AS-BI-phosphohydrolase, α-glucosidase, β-glucosidase, *N*-acetyl-β-glucosaminidase, α-mannosidase, and β-fucosidase and weakly positive for alkaline phosphatase, esterase (C4), valine arylamidase, cystine arylamidase, and acid phosphatase. It utilizes sole carbon sources D-maltose, sucrose, β-methyl-D-glucoside, *N*-acetyl-β-D-mannosamine, a-D-glucose, D-galactose, L-arginine, D-glucuronic acid, quinic acid, L-lactic acid, and L-malic acid. The predominant fatty acids (>10%) were iso-C_16:0_ and C_12:0_. The major polar lipid comprised diphosphatidylglycerol, phosphatidylglycerol, phosphatidylethanolamine, phosphatidylinositol mannoside, phosphatidylinositol, glycerol lipid, and six unidentified phospholipids. The cell-wall peptidoglycan contains *LL*-2,6-diaminopimelic acid, and the whole-cell sugars were galactose, glucose, xylose, and ribose. The predominant menaquinones were MK-10(H_4_) and MK-10(H_6_).

The type strain, SCSIO 64649^T^ (MCCC 1K06255^T^ = VKM Ac-2908^T^), was isolated from the stony coral *Favites* sp. collected from the South China Sea off the Luhuitou peninsula, Sanya, Hainan province, China. The complete genome of SCSIO 64649^T^ was composed of one linear chromosome 6,629,020 bp long with a G + C content of 73.6%, a total of 5,774 genes, and 32 biosynthetic gene clusters. The genome sequences for strains SCSIO 64649^T^ and SCSIO 03032 have been deposited to GenBank under accession numbers CP084541 and CP021121, respectively. The 16S rRNA gene sequences of strains SCSIO 64649^T^ and SCSIO 03032 have been deposited to GenBank under accession numbers MZ889118 and JN798514, respectively.

## Data Availability Statement

The datasets presented in this study can be found in online repositories. The names of the repository/repositories and accession number(s) can be found in the article/Supplementary Material.

## Author Contributions

SS and XT conceived and designed the study. SS and LC carried out all the experiments. LM and QL performed the LC/MS-based identification. KZ and QZ ran the bioinformatics analysis. SS prepared the manuscript. XT and LL revised the manuscript. All authors reviewed and approved the manuscript.

## Conflict of Interest

The authors declare that the research was conducted in the absence of any commercial or financial relationships that could be construed as a potential conflict of interest.

## Publisher’s Note

All claims expressed in this article are solely those of the authors and do not necessarily represent those of their affiliated organizations, or those of the publisher, the editors and the reviewers. Any product that may be evaluated in this article, or claim that may be made by its manufacturer, is not guaranteed or endorsed by the publisher.

## Footnotes

1
https://www.ezbiocloud.net

2
http://enve-omics.ce.gatech.edu/

## Abbreviations

DPG: diphosphatidylglycerol
PG: phosphatidylglycerol
PE: phosphatidylethanolamine
GL: glycerol lipid
PIM: phosphatidylinositol mannoside
PI: phosphatidylinositol
PL: unidentified phospholipid
L, unidentified lipid. R2A: Reasoner’s 2 agar
TSA: tryptic soy agar
NA: nutrient agar
ISP: International *Streptomyces* Project medium
ANI: average nucleotide identity
dDDH: digital DNA–DNA hybridization high-performance liquid chromatography (HPLC)
BGC: biosynthetic gene cluster
PKS: polyketide synthase
NRPS: non-ribosomal peptides synthase
RiPPs: ribosomally synthesized and post-translationally modified peptides.
