# Supplementary material for: Streptomyces marincola sp. nov., a Novel Marine Actinomycete, and Its Biosynthetic Potential of Bioactive Natural Products
Source: Front Microbiol. 2022 Apr 28;13:860308. doi: 10.3389/fmicb.2022.860308 (PMC9096227; doi:10.3389/fmicb.2022.860308)
Supplement: Supplementary file 1 [file Data_Sheet_1.docx]

**Supplementary material**

***Strepomyces marincola* sp. nov., A Novel Marine Actinomycete and its Biosynthetic Potential of Bioactive Natural Products**

**Song-Biao Shi^1,3^, Lin-Qing Cui^1,3^, Kun Zhang^1,3^, Qi Zeng^1,3^, Qing-lian Li^1,2^, Liang Ma^1^, Li-Juan Long^1,2*^, Xin-Peng Tian^1,2*^**

**^1^CAS Key Laboratory of Tropical Marine Bio-resources and Ecology; Guangdong Key Laboratory of Marine Materia Medica; CAS RNAM Center for Marine Microbiology; Sanya Institute of Oceanology, SCSIO; South China Sea Institute of Oceanology, Chinese Academy of Science, Guangzhou, Guangdong 510301, China**

**^2^Southern Marine Science and Engineering Guangdong Laboratory (Guangzhou)，511458, China**

**^3^University of Chinese Academy of Sciences, Beijing 100049, China**

***Authors for correspondence:**

**Xin Peng Tian**

**Tel / Fax: +86 20 89023378 E-Mail: xinpengtian@scsio.ac.cn**

**Li Juan Long**

**Tel / Fax: +86 20 89023121 E-Mail: longlj@scsio.ac.cn**

**Table. S1** The genome features of SCSIO 64649^T^, SCSIO 03032 and *S. specialis* DSM 41924^T^.

| Type | SCSIO 64649^T^ | SCSIO 03032 | DSM 41924^T^ |
| --- | --- | --- | --- |
| Genome size (bp) | 6,629,020 | 6,287,975 | 5,859,524 |
| Genes (total) | 5774 | 5488 | 5550 |
| Genes (protein-coding) | 5567 | 5294 | 5085 |
| Pseudo Genes (total) | 133 | 166 | ND |
| tRNAs | 56 | 55 | 53 |
| rRNAs | 15 | 15 | 64 |
| CRISPR arrays | 10 | 12 | 10 |
| G+C content (%) | 73.6 | 73.5 | 72.6 |

ND**, No data.**

**Table. S2** The genomic islands were identified in the genome of SCSIO 64649^T^ and SCSIO 03032, respectively.

| SCSIO 64649^T^ | | | | SCSIO 03032 | | | |
| --- | --- | --- | --- | --- | --- | --- | --- |
| Genomic Island | Start | End | Length  (bp) | Genomic Island | Start | End | Length  (bp) |
| 1 | 2090517 | 2099389 | 8872 | 1 | 1002858 | 1020489 | 17631 |
| 2 | 2823053 | 2833974 | 10921 | 2 | 1910086 | 1920303 | 10217 |
| 3 | 3113980 | 3124045 | 10065 | 3 | 2310054 | 2317214 | 7160 |
| 4 | 3178764 | 3198070 | 19306 | 4 | 3069476 | 3080130 | 10654 |
| 5 | 3516922 | 3526362 | 9440 | 5 | 3354096 | 3386624 | 32548 |
| 6 | 3599780 | 3620222 | 20442 | 6 | 5955934 | 6007724 | 51790 |
| 7 | 3639097 | 3649131 | 10034 | 7 | 6084694 | 6131596 | 46902 |
| 8 | 4228742 | 4247990 | 19248 |  |  |  |  |
| 9 | 6070815 | 6079710 | 8895 |  |  |  |  |
| 10 | 6491180 | 6502891 | 11711 |  |  |  |  |
| 11 | 6612029 | 6623545 | 11516 |  |  |  |  |

**Table. S3** The information of prophages was found in SCSIO 64649^T^ from the genome sequence.

| Strains | Number | Length | Category | Closest phage | Gene number |
| --- | --- | --- | --- | --- | --- |
| SCSIO 64649^T^ | 1 | 29743 | Ambiguous | *Streptomyces* phage Daudau | 22 |
|  | 2 | 25840 | Ambiguous | *Cyanophage* Syn2 | 24 |
|  | 3 | 10724 | Active | *Mycobacterium* phage Hammy | 8 |
| SCSIO 03032 | 1 | 32351 | Ambiguous | *Cyanophage* Syn2 | 101 |
|  | 2 | 16894 | Active | *Xanthomonas* phage Xoo-sp2 | 74 |
|  | 3 | 16690 | Ambiguous | *Pseudomonas* phage DL60 | 72 |

**Table S4** Cultural characteristics of strains SCSIO 64649^T^ and SCSIO 03032 at 28℃ for 14 days.

| Agar medium | SCSIO 64649^T^ | | | | | SCSIO 03032 | | | | |
| --- | --- | --- | --- | --- | --- | --- | --- | --- | --- | --- |
|  | Growth | Aerial mycelium | | Substrate mycelium | Soluble  pigment | Growth | Aerial mycelium | | Substrate mycelium | Soluble  pigment |
|  |  | Production | Color |  |  |  | Production | Color |  |  |
| ISP medium 1 | + | None | White | Light olive brown | None | + | None | White | Light olive brown | None |
| ISP medium 2 | +++ | Abundant | White | Dark olive brown | Black | +++ | Abundant | White | Dark olive brown | Black |
| ISP medium3 | + | None | White | White | None | + | None | White | White | None |
| ISP medium 4 | ++ | Moderate | White | olive brown | None | ++ | Moderate | White | olive brown | None |
| ISP medium 5 | ++ | Moderate | White | White grey | None | ++ | Moderate | White | White grey | None |
| ISP medium 6 | + | None | White | White | None | + | None | White | White | None |
| ISP medium 7 | +++ | Moderate | White | Greyish yellow | Black | +++ | Moderate | White | Greyish yellow | Black |
| Nutrient agar | +++ | Abundant | White | Yellow | None | +++ | Abundant | White | Yellow | None |
| Czapek’ s Solution agar | ++ | Moderate | White | Beige | None | ++ | Moderate | White | Beige | None |
| TSA | ++ | Moderate | Light red | Brown beige | None | ++ | Moderate | Light red | Brown beige | None |
| 2216E | +++ | Abundant | White | olive brown | red | +++ | Abundant | White | olive brown | red |

**Table. S5.** Phenotypic properties that distinguish isolates SCSIO 64649^T^ from their closest strain. +, positive; –, negative; w, weakly positive.

| Type | SCSIO 64649^T^ | SCSIO 03032 | DSM 41924^T^ |
| --- | --- | --- | --- |
| Enzyme activity in API ZYM: |  |  |  |
| Alkaline phosphatase | w | + | + |
| Esterase lipase (C8) | – | – | w |
| Lipase (C14) | + | + | w |
| Acid phosphatase | w | – | + |
| Naphthol-AS-BI-phosphohydrolase | + | w | + |
| *α*-glucosidase | + | – | w |
| *β*-glucosidase | + | – | – |
| N-acetyl-b-glucosaminidase | + | – | – |
| *α*-mannosidase | + | – | – |
| *β*-fucosidase | + | – | – |

**Table. S6** Comparing the biosynthetic gene clusters similarity in SCSIO 64649^T^ (#1-32) and SCSIO 03032 (#33-37).

| Cluster | Type | Most similar known cluster | SCSIO 64649^T^  (similarity) | SCSIO 03032  (similarity) | Unique BGCs |
| --- | --- | --- | --- | --- | --- |
| # 1 | Indole | Spiroindimicins/indimicins/lynamicins | 100 % | 100 % |  |
| # 2 | Terpene | Unknown | ND | ND |  |
| # 3 | Type I PKS | Piericidin A1 | 100 % | 100 % |  |
| # 4 | Type I PKS | Salinomycin | 6 % | 6 % |  |
| # 5 | Type II PKS, NRPS | Mayamycin | 68 % | — | Unique |
| # 6 | Siderophore | Desferrioxamine E | 50 % | 50 % |  |
| # 7 | Ectoine | Ectoine | 100 % | 100 % |  |
| # 8 | Thiopeptide, terpene | Lactazole | 33 % | 33 % |  |
| # 9 | Type I PKS | Streptazone E | 66 % | 66 % |  |
| # 10 | Lanthipeptide | AmfS | 80 % | 80 % |  |
| # 11 | Melanin | Grixazone | 38 % | 30 % |  |
| # 12 | NRPS | Unknown | ND | ND |  |
| # 13 | Type I PKS | Heronamides | 100 % | 100 % |  |
| # 14 | Lanthipeptide | Unknown | ND | — | Unique |
| # 15 | Type I PKS | Stambomycins | 16 % | 16 % |  |
| # 16 | Terpene | Isorenieratene | 37 % | 37 % |  |
| # 17 | Lanthipeptide | Unknown | ND | — | Unique |
| # 18 | Lanthipeptide | Unknown | ND | ND |  |
| # 19 | Terpene | Geosmin | 100 % | 100 % |  |
| # 20 | Type II PKS | Mayamycin | 54 % | 54 % |  |
| # 21 | Terpene | Carotenoid | 27 % | 27 % |  |
| # 22 | Phenazine | Unknown | ND | — | Unique |
| # 23 | RRE-containing | Unknown | ND | ND |  |
| # 24 | Lassopeptide | Unknown | ND | — | Unique |
| # 25 | Siderophore | Unknown | ND | ND |  |
| # 26 | Terpene | Hopene | 30 % | 30 % |  |
| # 27 | Type III PKS | Flaviolin | 50 % | 50 % |  |
| # 28 | RRE-containing | Anantin C | 50 % | — | Unique |
| # 29 | RiPP-like | Unknown | ND | ND |  |
| # 30 | Type I PKS | Mediomiycin A | 36 % | 32 % |  |
| # 31 | Guanidinotides | Ketomemicins B3/B4 | 100 % | — | Unique |
| # 32 | Terpene | Unknown | ND | — | Unique |
| # 33 | Lanthipeptide | Unknown | — | ND | Unique |
| # 34 | NRPS-like | Unknown | — | ND | Unique |
| # 35 | Type I PKS | Lobosamides | — | 19 % | Unique |
| # 36 | Lassopeptide | Unknown | — | ND | Unique |
| # 37 | PKS-like | Unknown | — | ND | Unique |

ND, None data, — Non-existent gene cluster.

**Table. S7** Comparing the BGCs amino acid similarity of cluster #5 in strain SCSIO 64649^T^ with *may* gene cluster.

| **Cluster #5** | Size  (aa) | Proposed function | Mayamycin (identify/coverage) |
| --- | --- | --- | --- |
| LC193_03450 | 180 | SnoaL-like domain | 53/79 |
| LC193_03455 | 253 | short-chain dehydrogenase/reductase | — |
| LC193_03460 | 477 | NDP-hexose 2,3-dehydratase | 61/96 |
| LC193_03465 | 201 | dTDP-4-dehydrorhamnose 3,5-epimerase | — |
| LC193_03470 | 373 | DegT/DnrJ/EryC1/StrS aminotransferase | 78/100 |
| LC193_03475 | 496 | monooxygenase FAD-binding | 64/100 |
| LC193_03480 | 342 | dTDP-glucose 4,6-dehydratase | 72/92 |
| LC193_03485 | 416 | glycosyltransferase | — |
| LC193_03490 | 228 | Antibiotic biosynthesis monooxygenase |  |
| LC193_03495 | 296 | glucose-1-phosphate adenylyl/thymidylyltransferase | 74/88 |
| LC193_03500 | 559 | monooxygenase FAD-binding | 50/96 |
| LC193_03505 | 326 | cyclase/dehydrase | 69/96 |
| LC193_03510 | 279 | short-chain dehydrogenase/reductase | 83/92 |
| LC193_03515 | 86 | acyl carrier protein | 72/95 |
| LC193_03520 | 408 | Beta-ketoacyl synthase | 68/97 |
| LC193_03525 | 423 | Beta-ketoacyl synthase | 82/100 |
| LC193_03530 | 110 | Polyketide synthesis cyclase | 75/98 |
| LC193_03535 | 207 | AefR-like transcriptional repressor |  |
| LC193_03540 | 244 | response regulator | 60/92 |

— Non-existent gene similarity.

**Table. S8** Comparing the BGCs amino acid similarity of cluster #20 in strain SCSIO 64649^T^ with *may* gene cluster.

| **Cluster #20** | Size  (aa) | Proposed function | Mayamycin  (identify/ coverage) |
| --- | --- | --- | --- |
| LC193_16250 | 346 | Acyl transferase | — |
| LC193_16255 | 362 | 3-Oxoacyl-[acyl-carrier-protein (ACP)] synthase | — |
| LC193_16260 | 91 | acyl carrier protein | — |
| LC193_16265 | 384 | glycosyltransferase | — |
| LC193_16270 | 392 | glycosyltransferase | — |
| LC193_16275 | 334 | dTDP-glucose 4,6-dehydratase | 64/97 |
| LC193_16280 | 295 | glucose-1-phosphate thymidylyltransferase | 64/97 |
| LC193_16285 | 476 | monooxygenase FAD-binding | 52/100 |
| LC193_16290 | 315 | cyclase/dehydrase | 63/98 |
| LC193_16295 | 265 | short chain dehydrogenase | 77/96 |
| LC193_16300 | 404 | Beta-ketoacyl synthase | 64/100 |
| LC193_16305 | 424 | Beta-ketoacyl synthase | 75/100 |
| LC193_16310 | 107 | Polyketide synthesis cyclase | 62/99 |
| LC193_16315 | 509 | monooxygenase FAD-binding | 61/96 |
| LC193_16320 | 206 | dTDP-4-dehydrorhamnose 3,5-epimerase | — |
| LC193_16325 | 369 | DegT/DnrJ/EryC1/StrS aminotransferase family | 77/100 |
| LC193_16330 | 239 | methyltransferase | 48/97 |
| LC193_16335 | 334 | NAD dependent epimerase/dehydratase | — |
| LC193_16340 | 476 | NDP-hexose 2,3-dehydratase | 47/97 |
| LC193_16350 | 201 | response regulator | — |

— Non-existent gene similarity.

**Table. S9** The information of serine protease in strain SCSIO 64649^T^.

| Gene number | Function |
| --- | --- |
| SCSIO64649-liner_0166 | putative secreted subtilisin-like serine protease |
| SCSIO64649_liner_0167 | serine protease |
| SCSIO64649_liner_0221 | putative secreted subtilisin-like serine protease |
| SCSIO64649_liner_0482 | serine protease |
| SCSIO64649_liner_1086 | putative secreted serine protease |
| SCSIO64649_liner_1788 | serine protease |
| SCSIO64649_liner_2658 | putative serine protease |
| SCSIO64649_liner_3041 | secreted serine protease |
| SCSIO64649_liner_3271 | serine protease |
| SCSIO64649_liner_3389 | putative secreted subtilisin-like serine protease |
| SCSIO64649_liner_3415 | putative secreted subtilisin-like serine protease |
| SCSIO64649_liner_3597 | putative secreted subtilisin-like serine protease |
| SCSIO64649_liner_4038 | putative secreted subtilisin-like serine protease |
| SCSIO64649_liner_4083 | serine protease |
| SCSIO64649_liner_4389 | putative secreted subtilisin-like serine protease |
| SCSIO64649_liner_4764 | serine protease |
| SCSIO64649_liner_4769 | secreted serine protease |
| SCSIO64649_liner_4771 | secreted serine protease |
| SCSIO64649_liner_5053 | putative secreted subtilisin-like serine protease |
| SCSIO64649_liner_5167 | serine protease |
| SCSIO64649_liner_5254 | putative secreted subtilisin-like serine protease |

**Table S10** Comparing the eight genes amino acid similarity of *spm* gene cluster with strain SCSIO 03032.

| Genes | Gene function | Amino acids  (coverage) | Similarity (%) |
| --- | --- | --- | --- |
| *spmR* | transcriptional activator | 930/958 | 98.31 |
| *spmA* | ABC transporter | 519/549 | 94.54 |
| *spmO* | tryptophan oxidase | 476/485 | 98.14 |
| *spmD* | chromopyrrolic acid synthase | 1079/1100 | 98.09 |
| *spmP* | cytochrome P450 | 398/427 | 92.56 |
| *spmF* | flavin reductase | 194/196 | 98.98 |
| *spmH* | tryptophan halogenase | 508/513 | 99.03 |
| *spmX2* | monooxygenase | 443/448 | 98.88 |


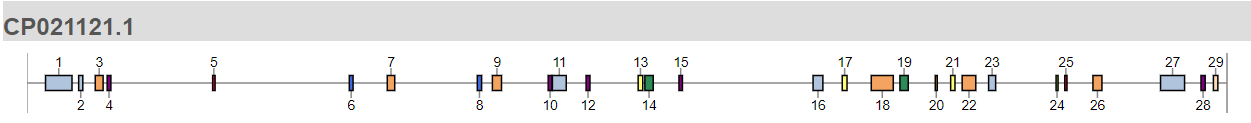

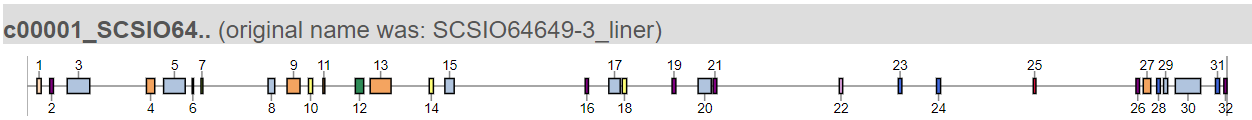


SCSIO 03032

SCSIO 64649^T^

**Fig S1.** Biosynthetic gene cluster maps in strains SCSIO 64649 and SCSIO 03032.


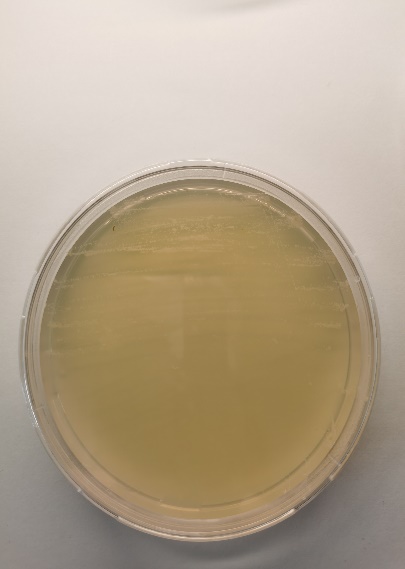


ISP1


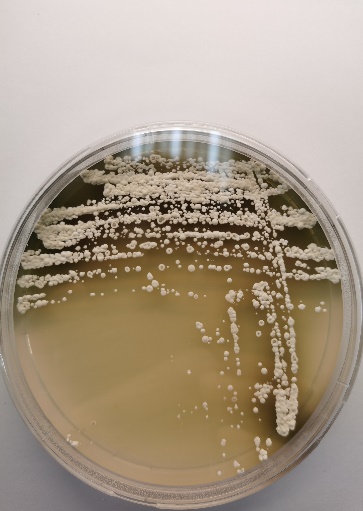


ISP2


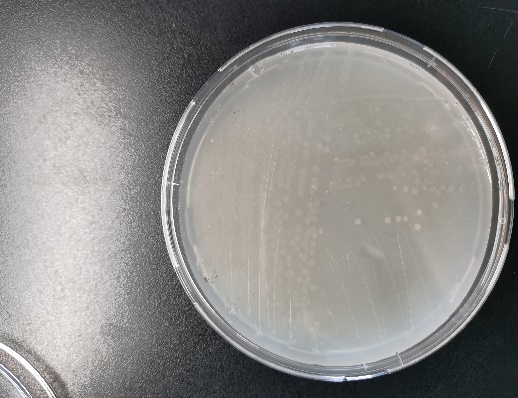


ISP4


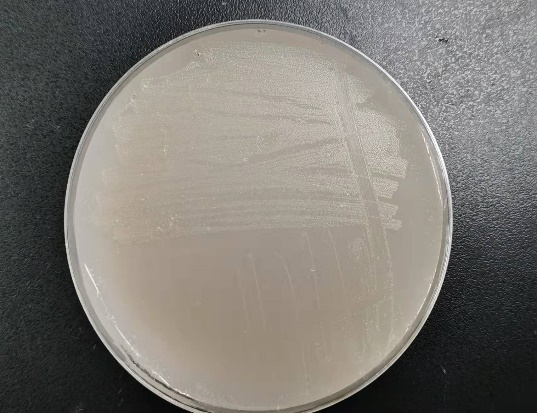


ISP3


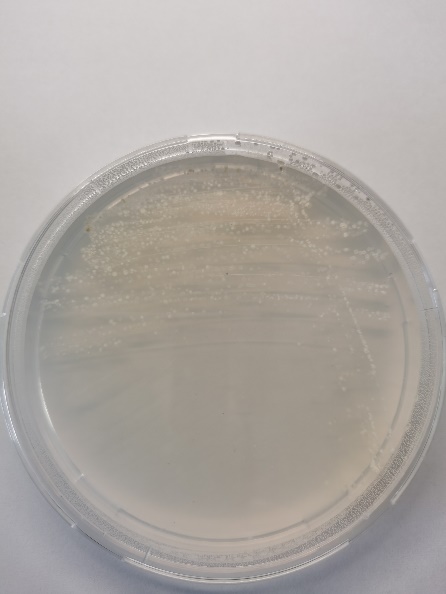


ISP5


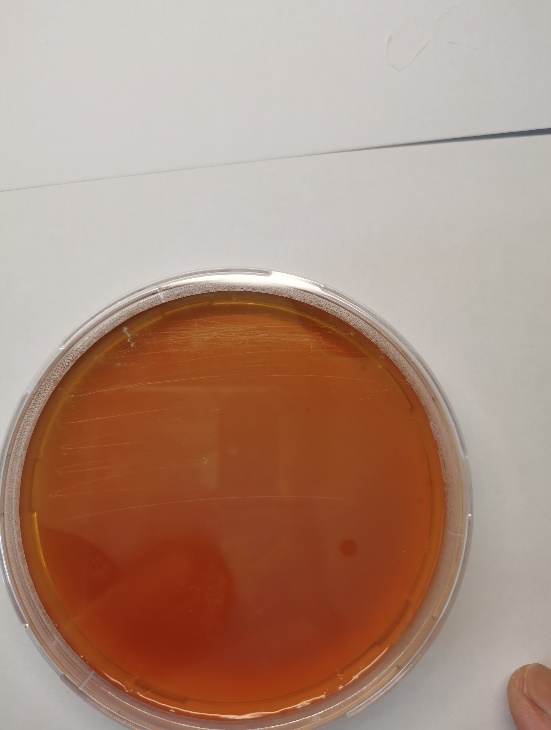


ISP6


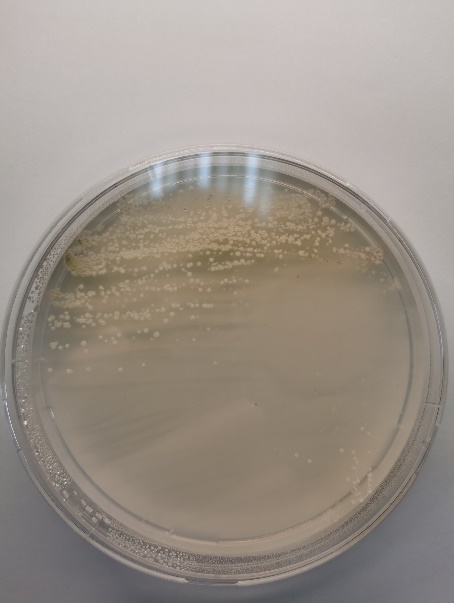


ISP7


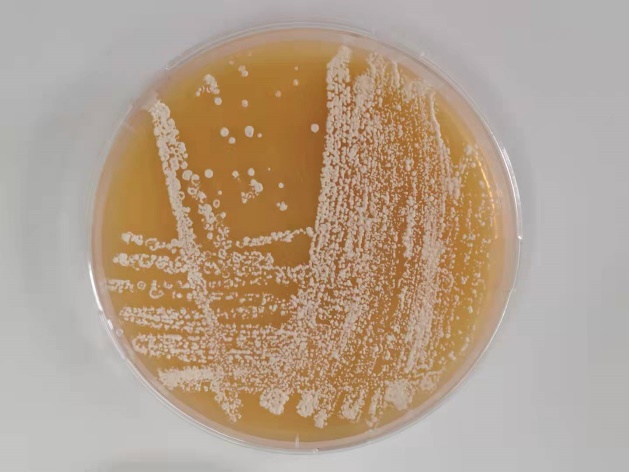


NA


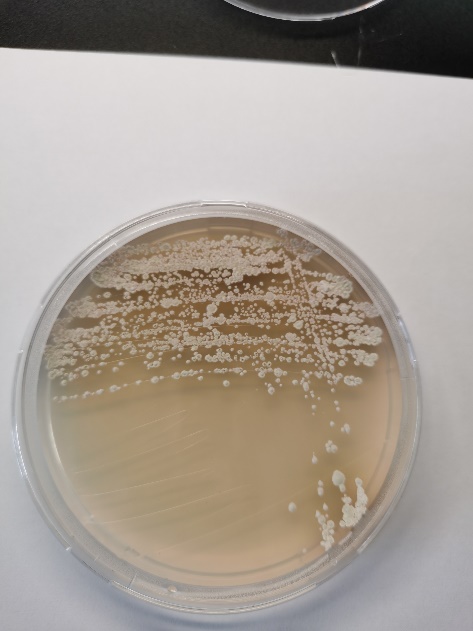


2216


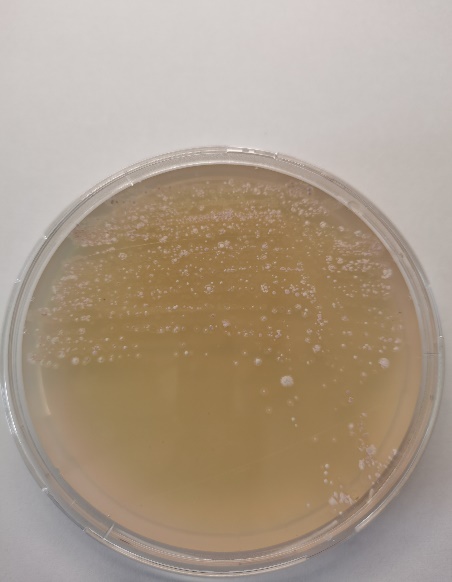


TSA

**Fig. S2** The growth conditions on different media of strain SCSIO 64649^T^.


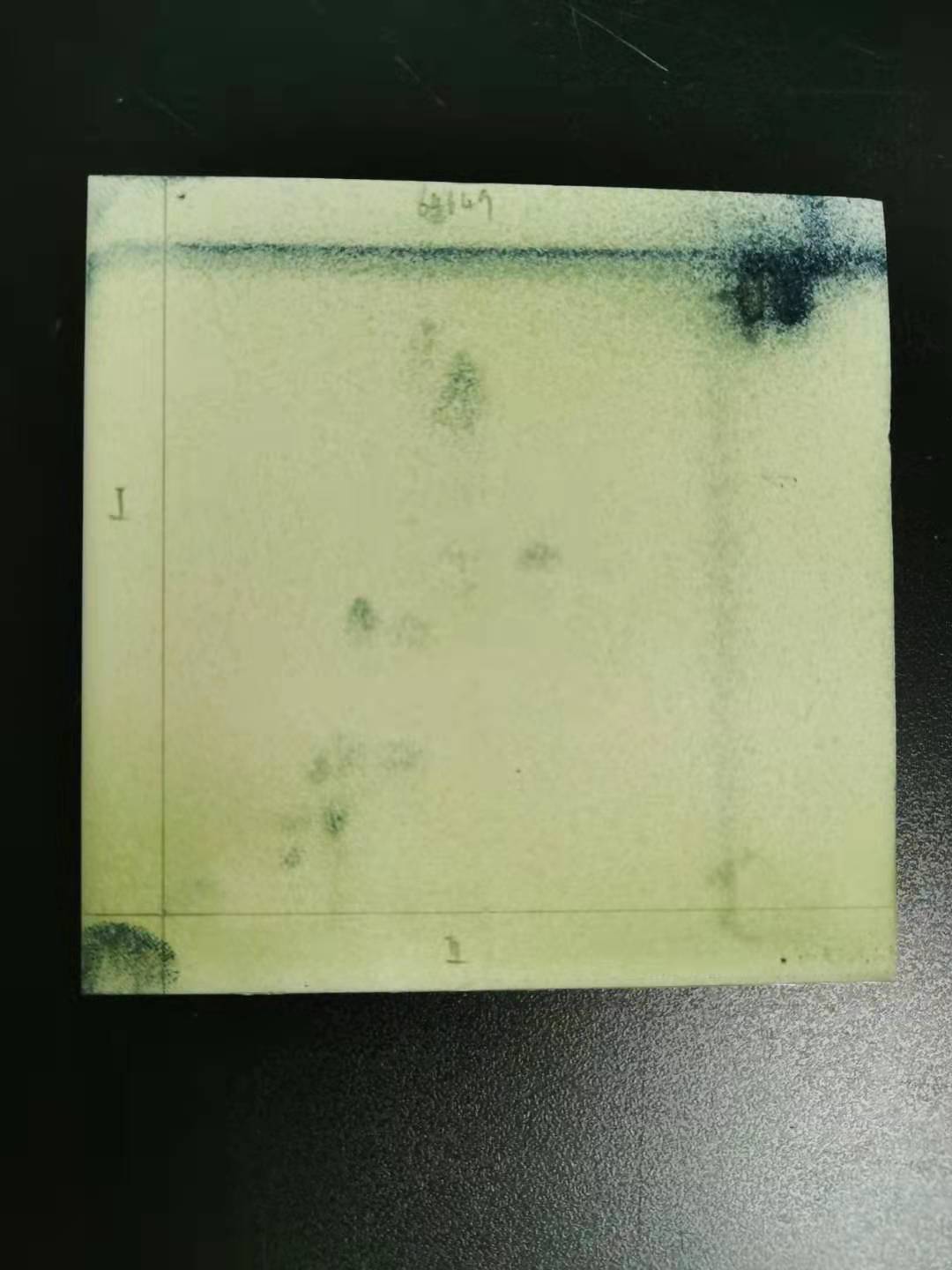


PL1

DPG

PE

PG

PL2

PL3

PL4

PI

PL5

PIM

PL6

GL


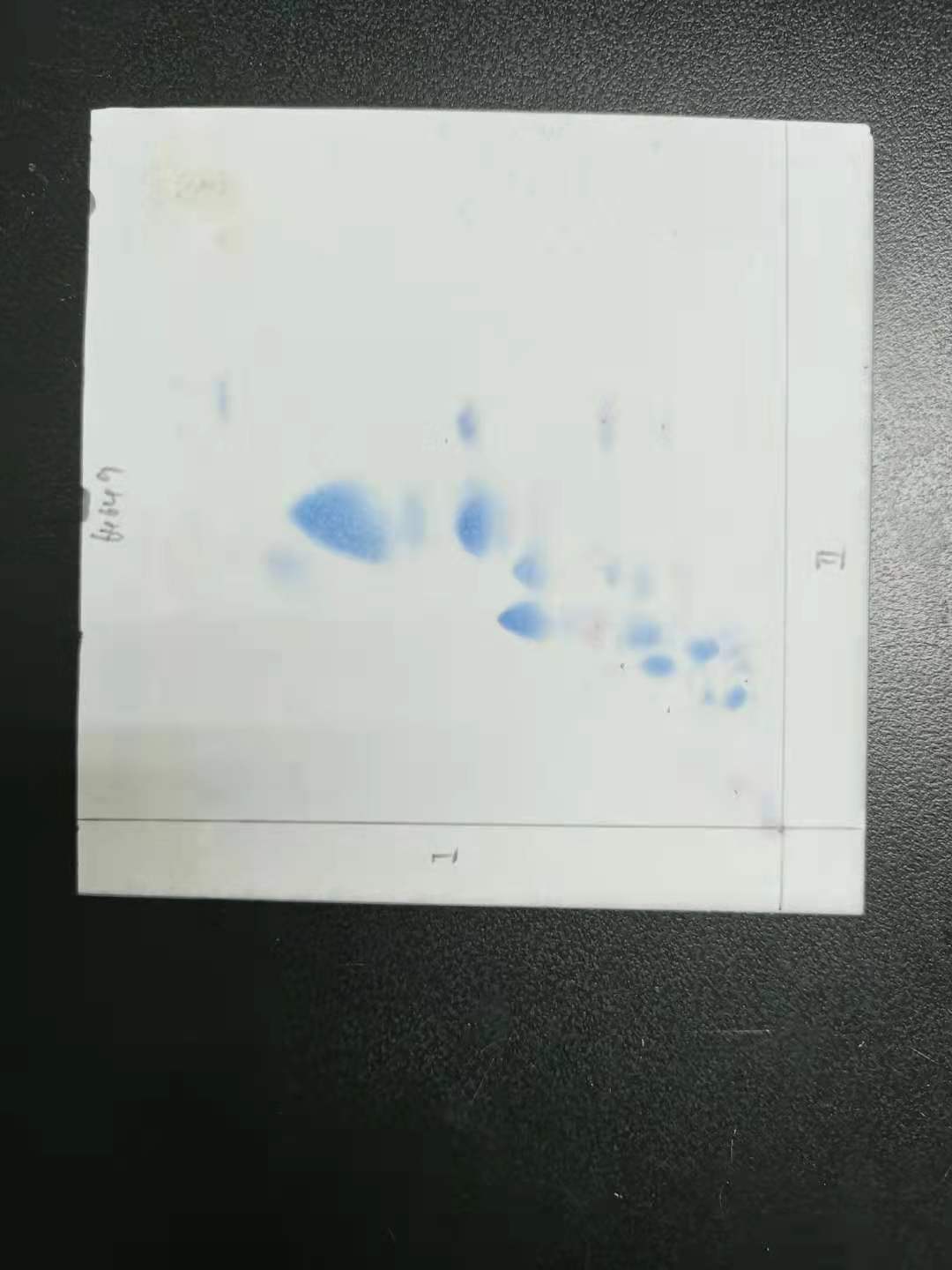


DPG

PL2

PI

PIM

PE

PL4

PL5

PG

PL3

PL6

PL1


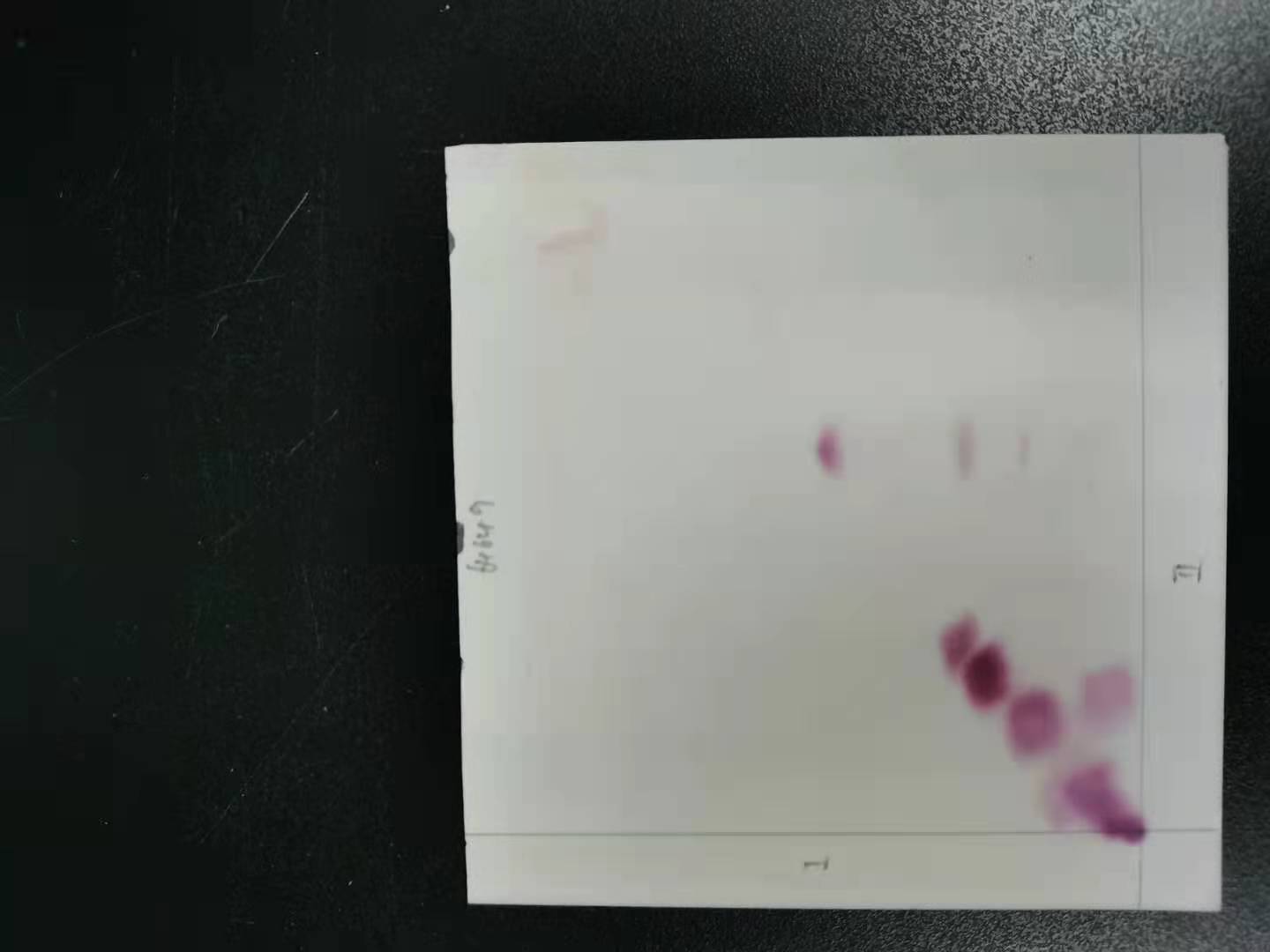


PE


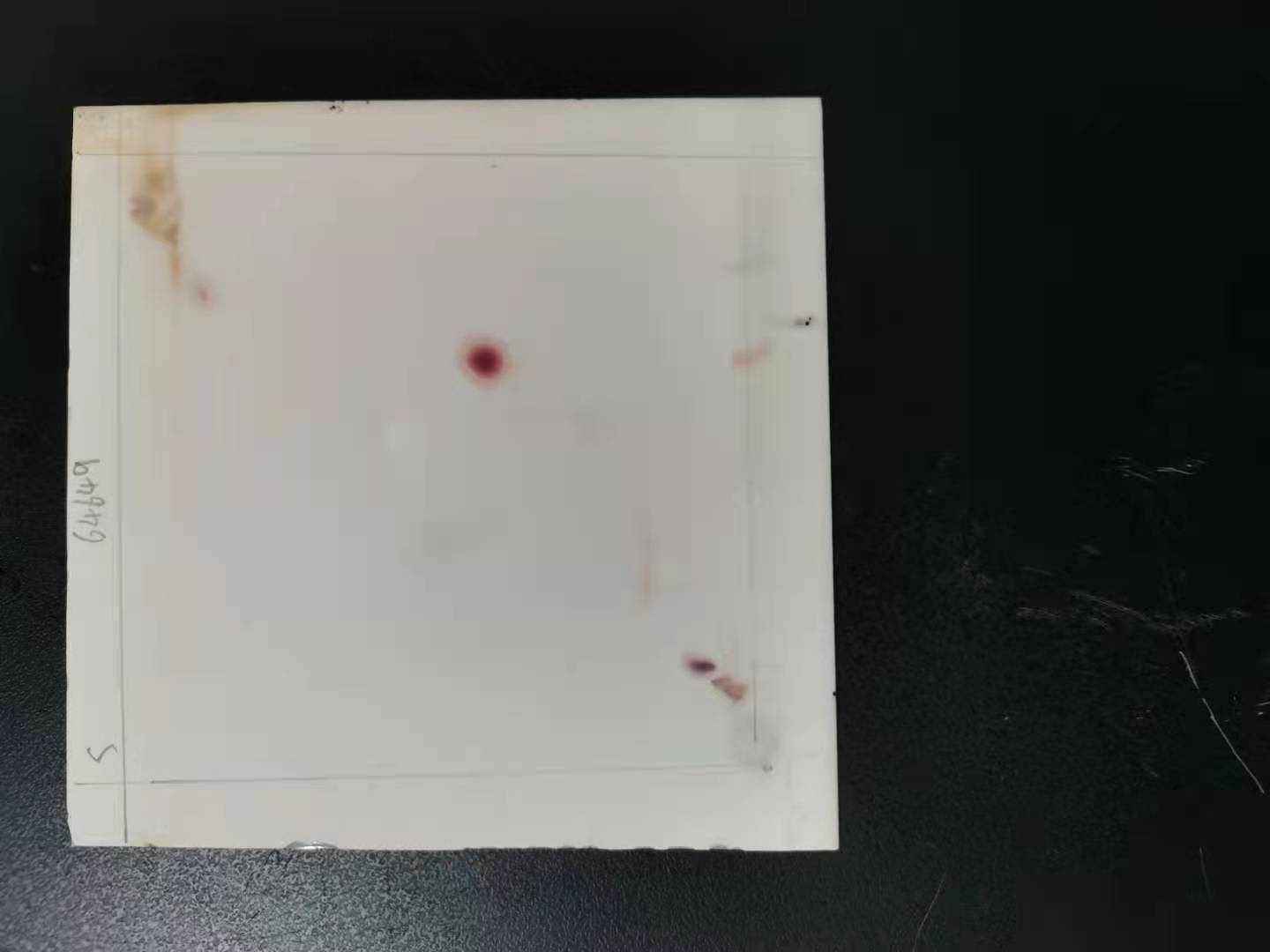


GL

PIM

**B**-64649

**A**-64649

**D**-64649

**C**-64649

**A**-41924

**B**-41924


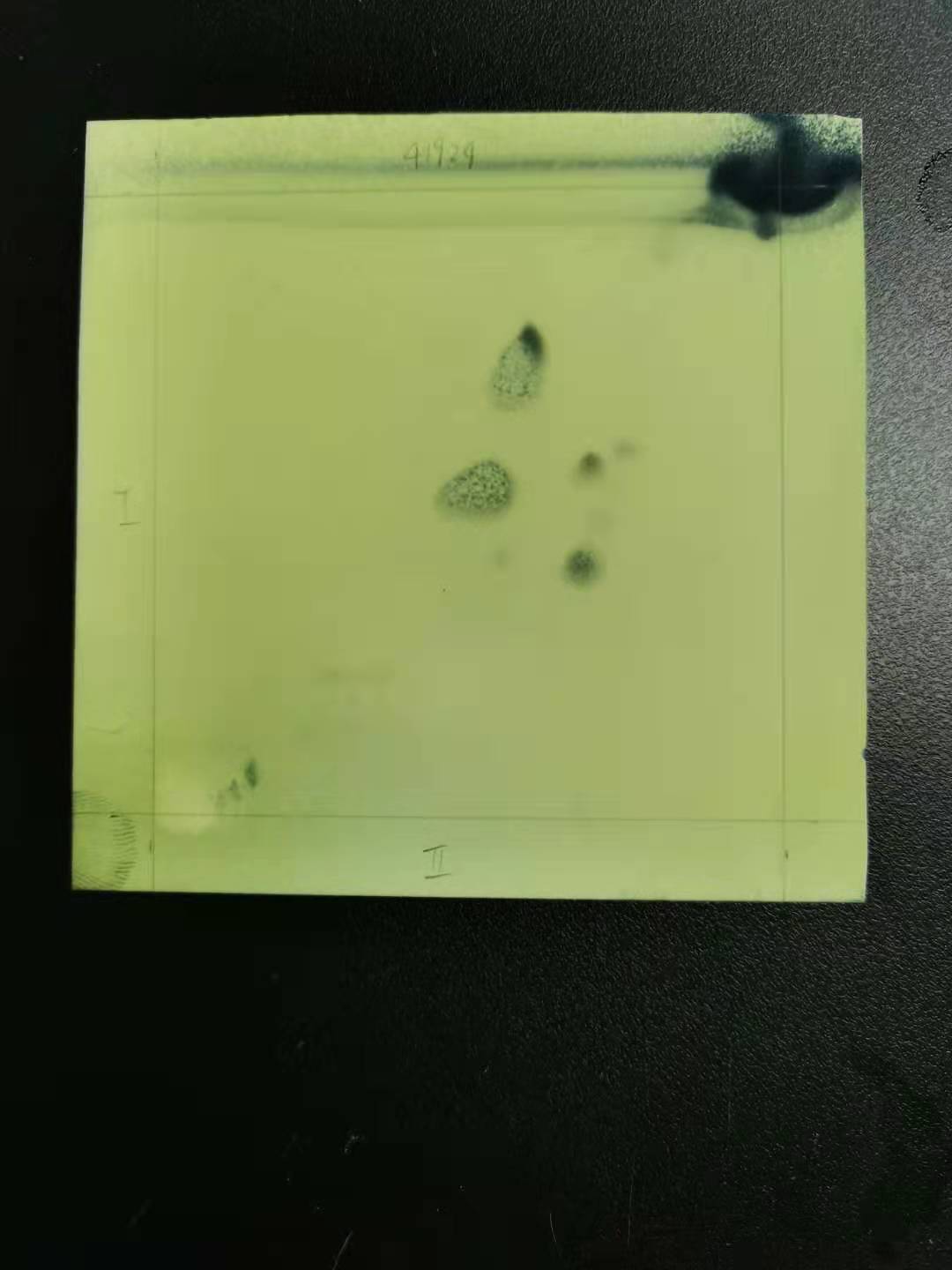


GL

PE

PI

PIM

DPG

L1

L2

PL2

PG

PL1


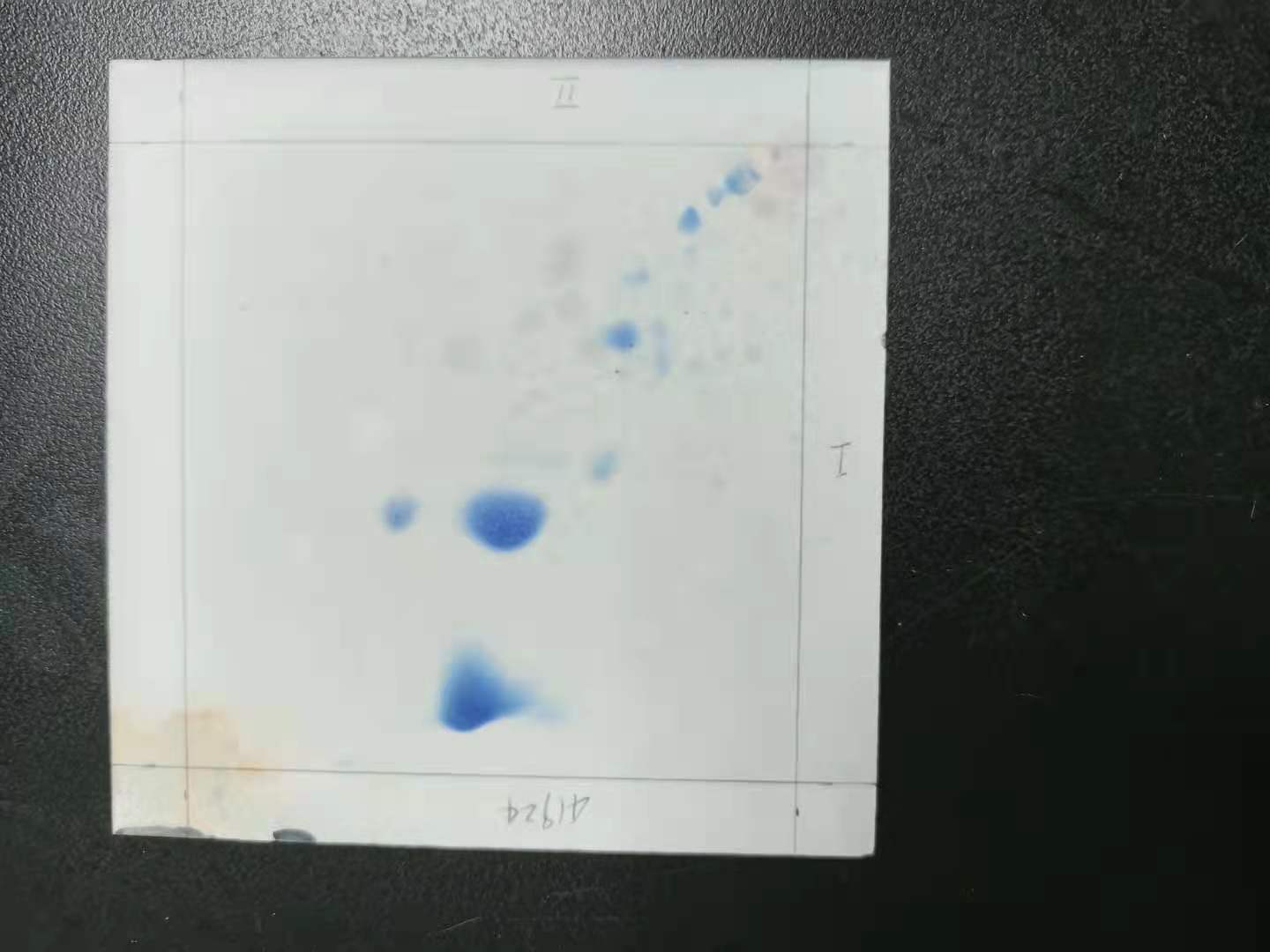


PG

PE

PI

PIM

PL1

DPG

PL2


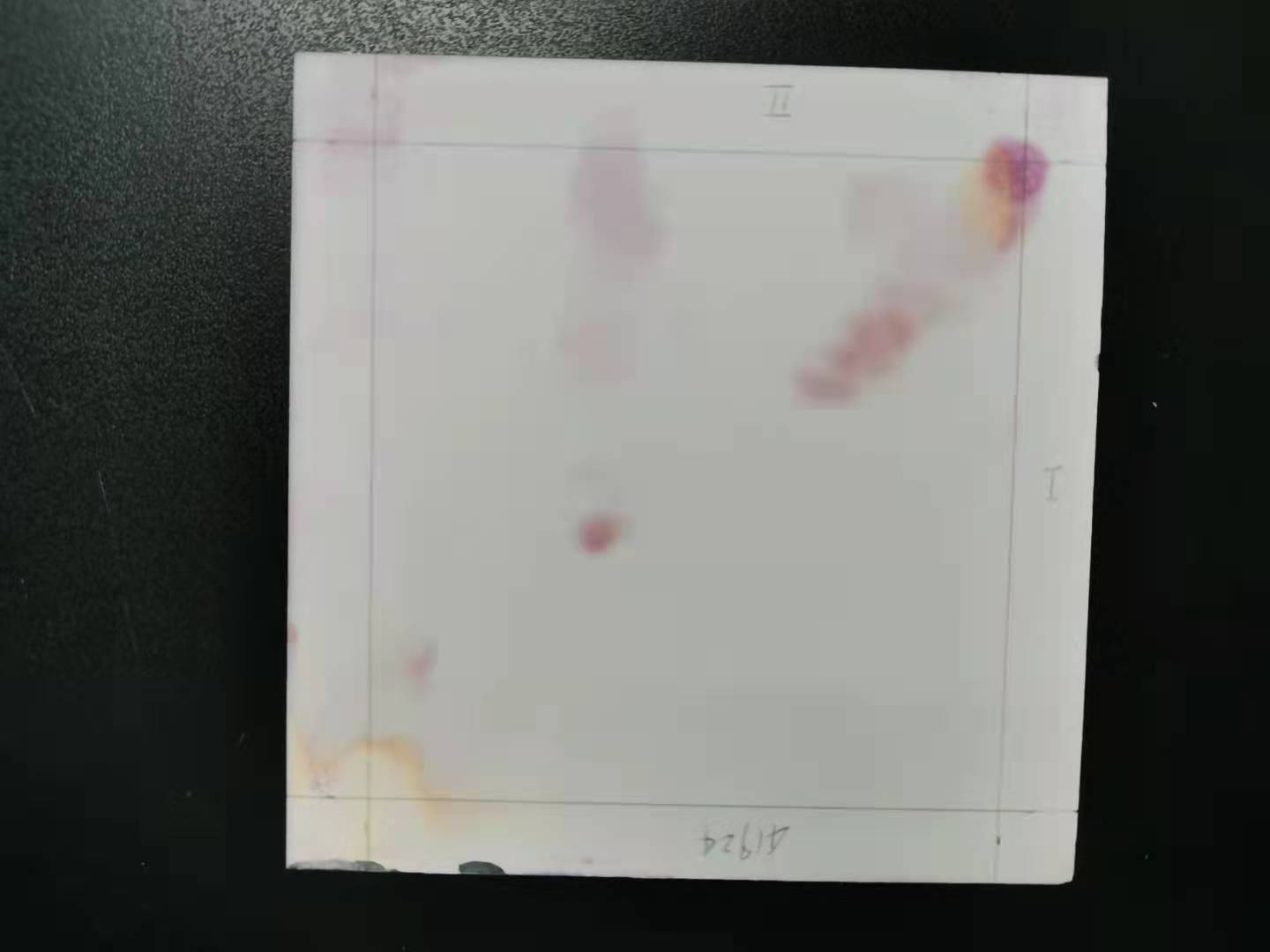


PE


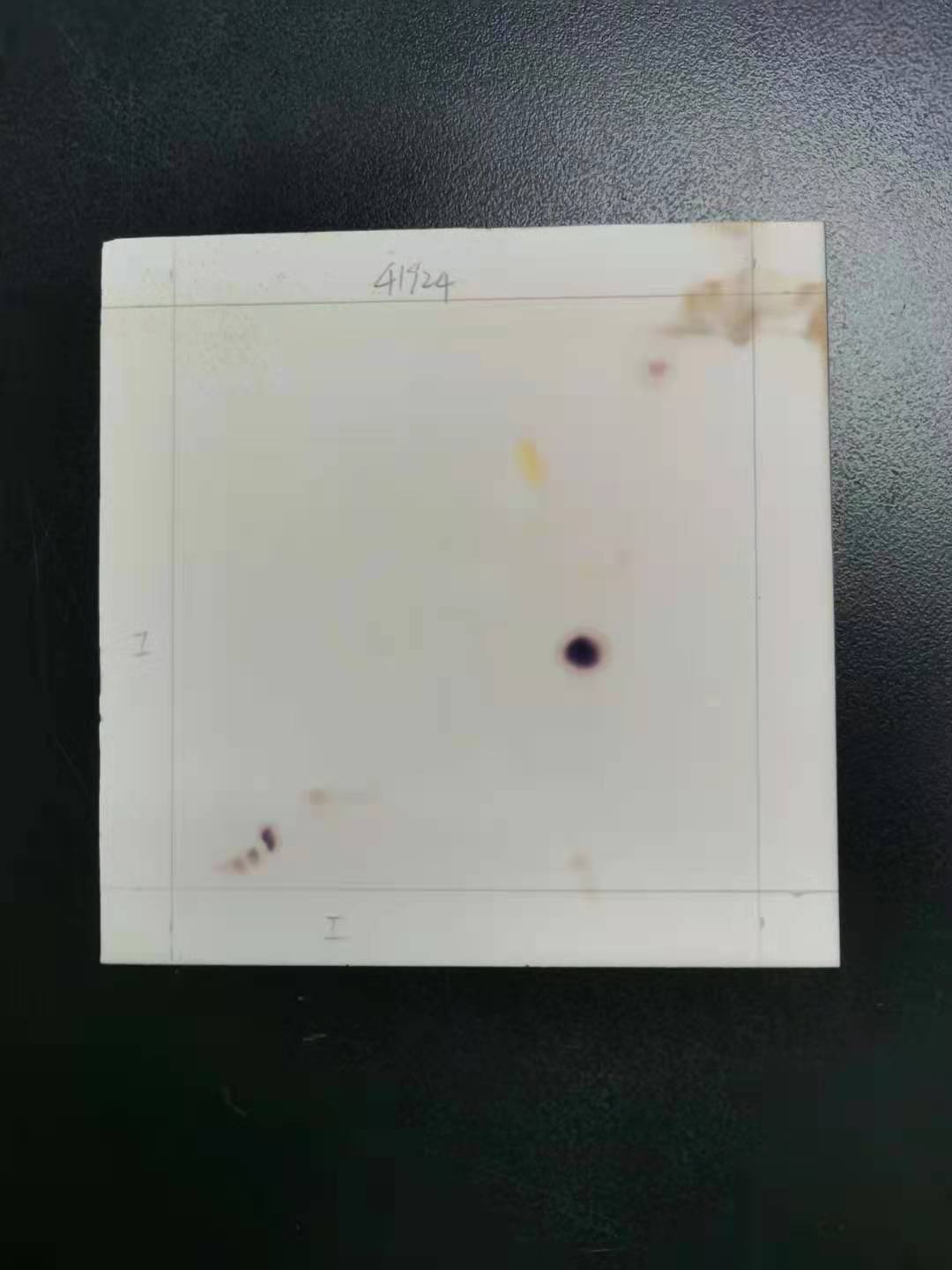


GL

PIM

**D**-41924

**C**-41924

**Fig. S3** Two-dimensional TLC patterns of the total polar lipids of strain SCSIO 64649^T^ and reference strain *Streptomyces specialis* DSM 41924^T^. The solvent systems were chloroform-methanol-water (65: 25: 4, v/v) for the first dimension and chloroform-acetic acid-methanol-water (80: 18: 12: 4, v/v) for the second dimension. The following spray reagents were A, molybdatophosphoric acid (for total lipids), B, molybdenum blue (for phospholipids), C, ninhydrin (for aminolipids) and D, 1-naphthol reagent. DPG, diphosphatidylglycerol; PG, phosphatidylglycerol; PE, phospatidylethanolamine; GL, glycerol lipids; PIM, phosphatidylinositol mannoside; PI, phosphatidylinositol, PL, unidentified phospholipid, L, unidentified lipid.


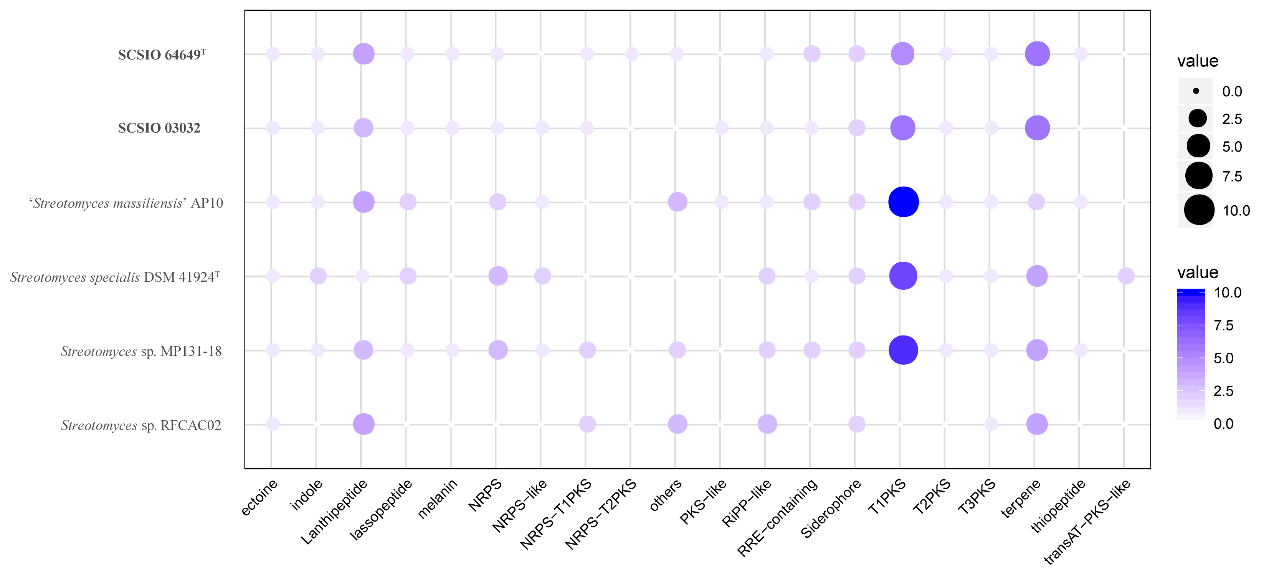


**Fig. S4** Biosynthetic gene clusters found in the genomes of representative isolates and their closest strains using AntiSMASH 6.0 software.


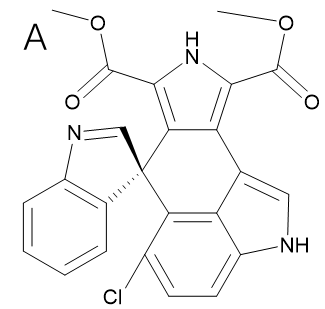

**B**

**C1**

**C2**

**C3**

**C4**

**D1**

**D2**

**D3**

**D4**

**E1**

**E2**

**E3**

**F**

**Fig. S5** The structures of predicted compounds based on biosynthetic gene clusters using PRISM 4. A, Compound encoded by gene cluster 1#; B, Compound encoded by gene cluster 3#; C1-C4, Compounds encoded by gene cluster 5#; D1-D4, Compounds encoded by gene cluster 13#; E1-E3, Compounds encoded by gene cluster 20#; F, Compound encoded by gene cluster 30#.


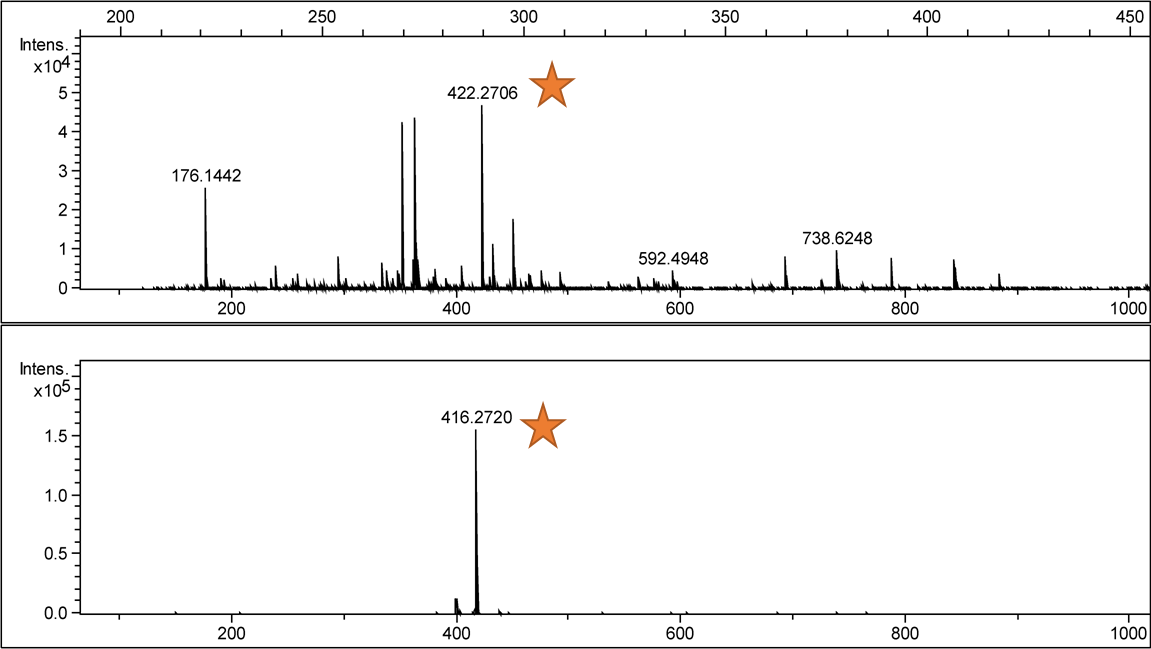


**Fig. S6** HR**-**ESI-MS spectrum of heronamide F and pieicidin A1 from the LC-MS analysis of the fermentation extract of strain SCSIO 64649^T^. The mass is consistent with the proposed structure of heronamide F and pieicidin A1 with m/z [M+H]^+^ =422.2706 (calculated 422.2708)and [M+H]^+^ =416.2720 (calculated 416.2722).


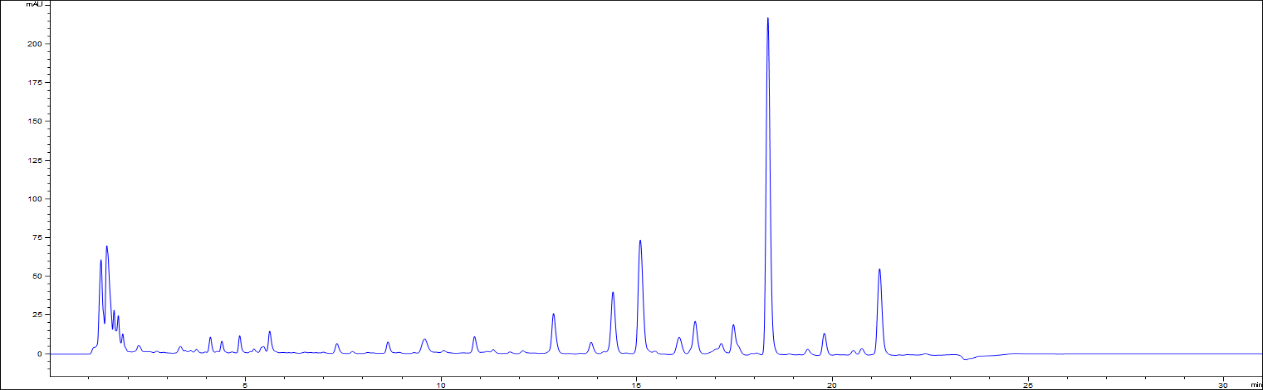

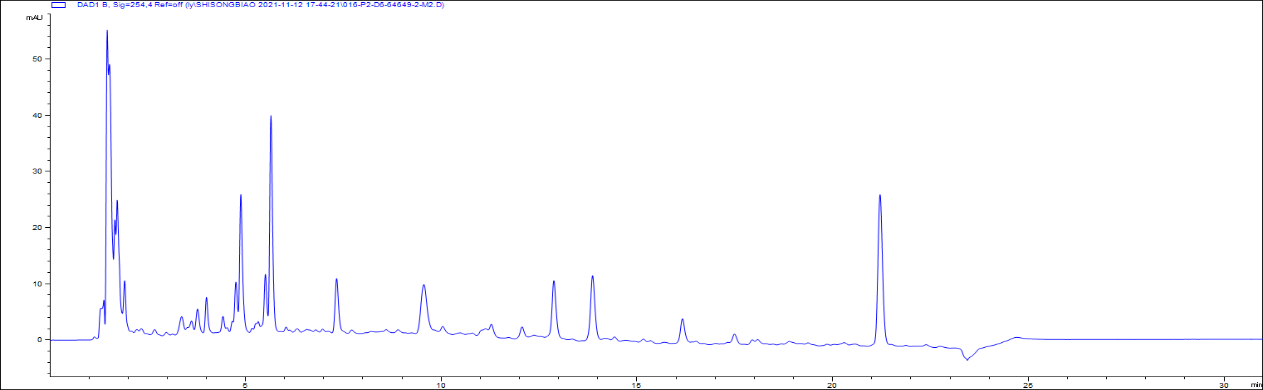


Pieicidin A1

Pieicidin A1

Spiroindimicin B

Spiroindimicin A

Lynamicin A/D

Heronamide F

Spiroindimicin A

Heronamide F

SCSIO 64649^T^

SCSIO 03032

**A**


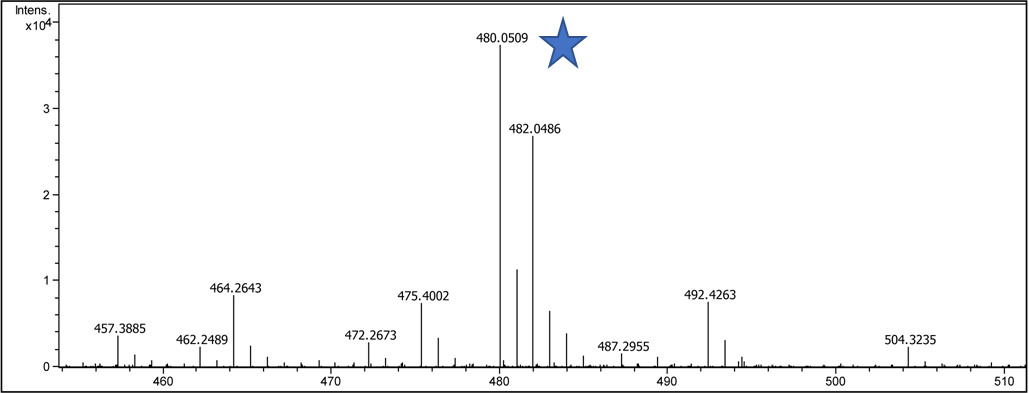


**B**

**Fig. S7** (A) LC-diode array detector (DAD) isoplot of the analyzed extract showing compounds produced. (B) HR**-**ESI-MS spectrum of spiroindimicin A from the LC-MS analysis of the fermentation extract of strain SCSIO 64649^T^. The mass is consistent with the proposed structure of spiroindimicin A with m/z [M+H]^+^ =480.0509 (calculated 480.0361).


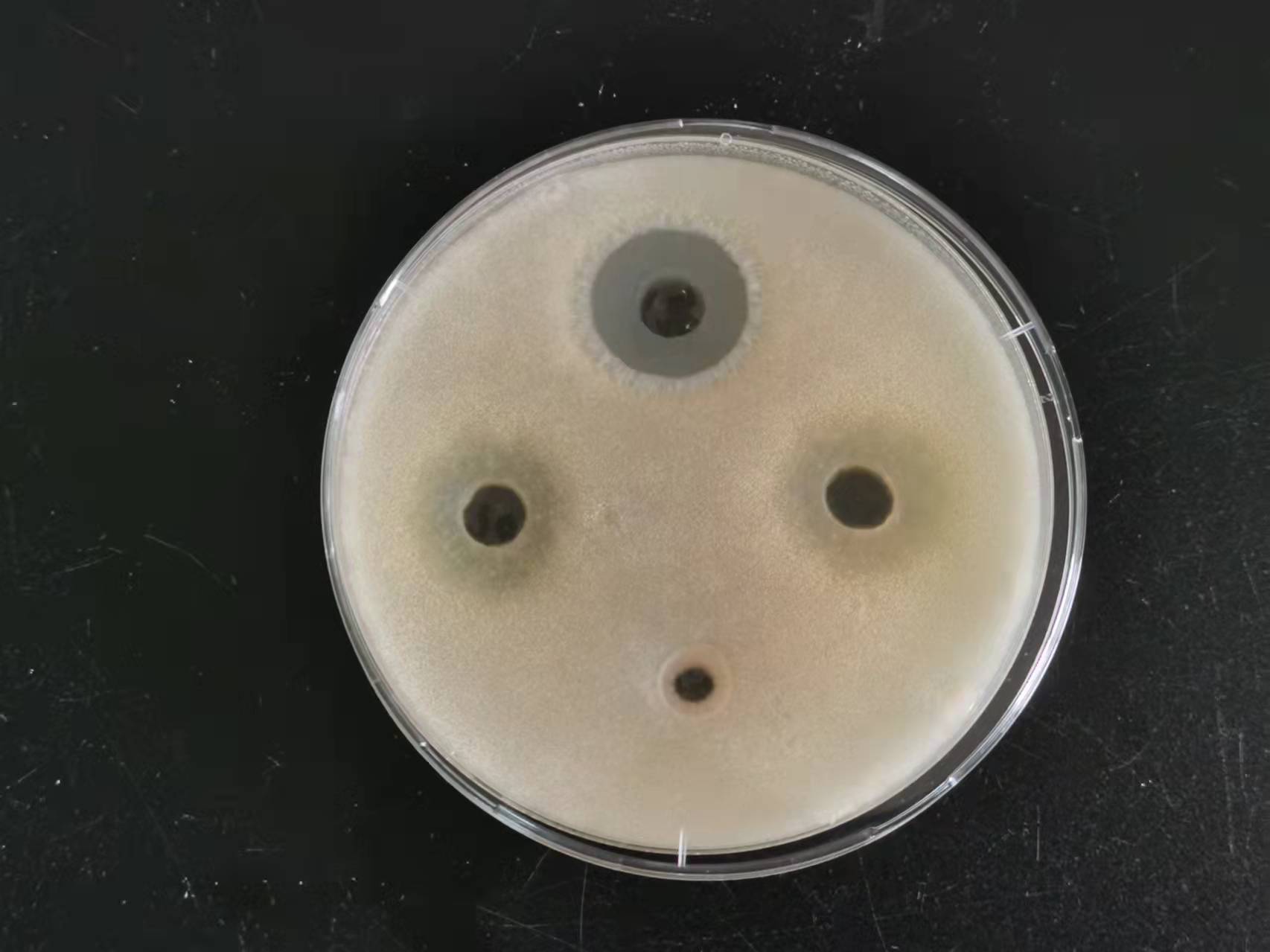

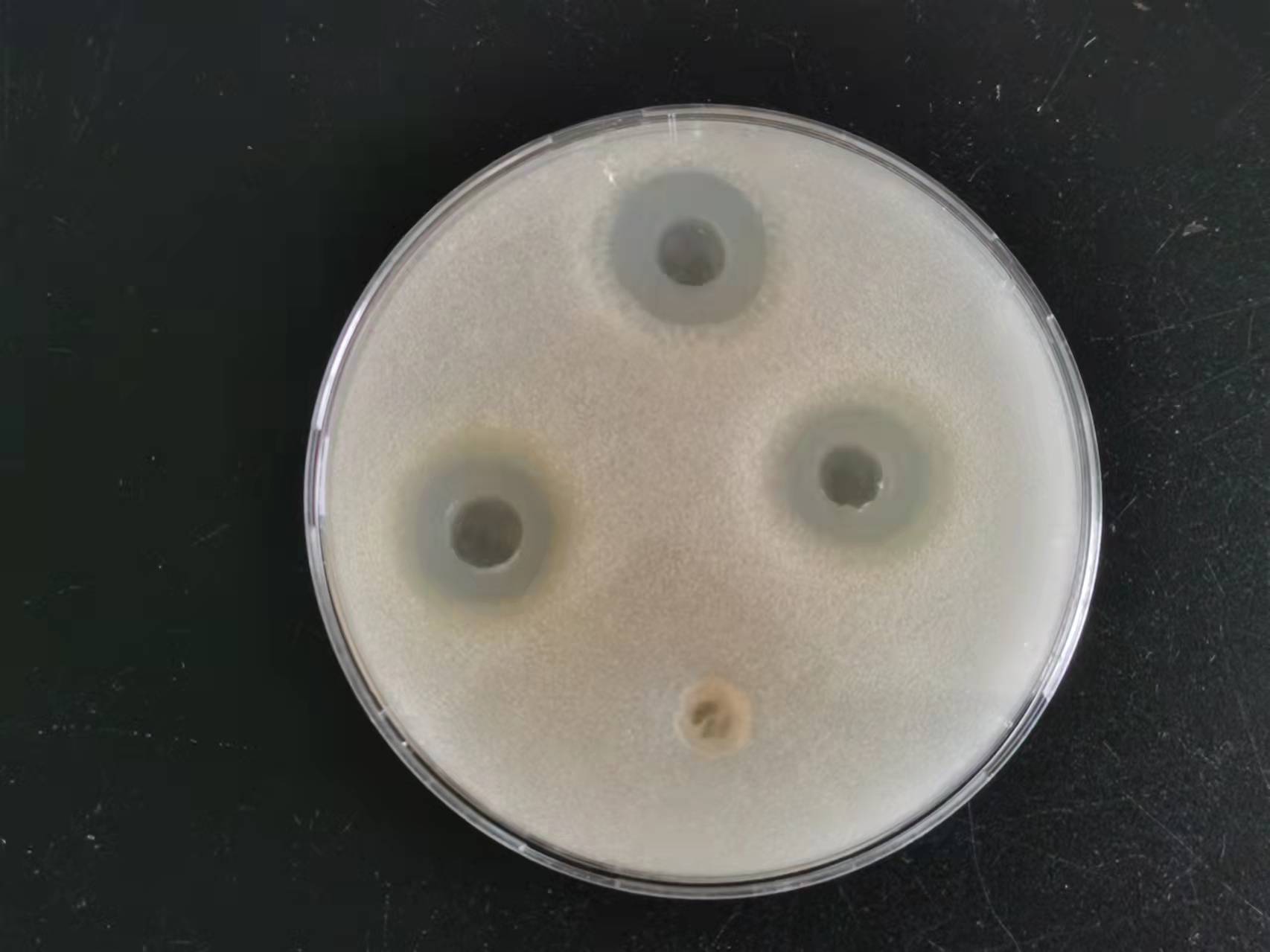


*Aspergillus niger*

Nystatin

Nystatin

64649

03032

64649

03032

ISP4#

ISP3#

DMSO

DMSO

**Fig. S8** Antifungal activity of the crude extracts from strains SCSIO 64649^T^ and SCSIO 03032 using two fermentation media with Nystatin (2.56 mg/mL) and DMSO as a positive and negative control.
